# Microbial Origin of Aquaponic Water Suppressiveness against *Pythium aphanidermatum* Lettuce Root Rot Disease

**DOI:** 10.3390/microorganisms8111683

**Published:** 2020-10-29

**Authors:** Gilles Stouvenakers, Sébastien Massart, Pierre Depireux, M. Haïssam Jijakli

**Affiliations:** Integrated and Urban Plant Pathology Laboratory, Gembloux Agro-Bio Tech, University of Liège, 5030 Gembloux, Belgium; sebastien.massart@uliege.be (S.M.); depireuxpierre@gmail.com (P.D.); mh.jijakli@uliege.be (M.H.J.)

**Keywords:** aquaponic, disease suppressive, *Pythium aphanidermatum*, lettuce, high-throughput sequencing, microorganism, bacteria, fungi

## Abstract

Aquaponic systems are an integrated way to produce fish and plants together with mutual benefits. Fish provide nutrients to plants on the one side, and plant nutrients uptake allow water reuse for fish on the other side. In this kind of system, the use of phytosanitary treatments to control plant pathogens is sensitive because of the risk of toxicity for fish present in the same water loop, especially coupled aquaponics. Among plant pathogens, *Pythium aphanidermatum* is a most problematic microorganism due to the Oomycete’s capacity to produce mobile form of dispersion (zoospores) in the recirculated water. Therefore, this study aimed at elucidating the potential antagonistic capacity of aquaponic water against *P. aphanidermatum* diseases. It was shown that aquaponic water presented an inhibitory effect on *P. aphanidermatum* mycelial growth in in vitro conditions. The same result was observed when lettuce plants growing in aquaponic water were inoculated by the same plant pathogen. Aquaponic lettuce was then compared to lettuce grown in hydroponic water or complemented aquaponic water (aquaponic water plus mineral nutrients). The disease was suppressed in the presence of aquaponic water, contrary to lettuce grown in hydroponic water or complemented aquaponic water. Root microbiota were analyzed by 16S rDNA and ITS Illumina sequencing to determine the cause of this aquaponic suppressive action. It was determined that the diversity and the composition of the root microbiota were significantly correlated with the suppressive effect of aquaponic water. Several taxa identified by metabarcoding were suspected to be involved in this effect. Moreover, few of these microorganisms, at the genus level, are known to have an antagonistic effect against *P. aphanidermatum*. These innovative results indicate that aquaponic water could be an interesting and novel source of antagonistic agents adapted to control *P. aphanidermatum* diseases in soilless culture.

## 1. Introduction

In one loop (i.e., coupled) aquaponic systems, the control of plant pathogens is complex because of the simultaneous presence of fish and nitrifying bacteria in the same loop as plants. Indeed, the addition of chemical agents (e.g., disinfecting agents) and/or pesticides in the irrigation system could be toxic for both fish and nitrifying bacteria [1]. In Europe, pesticides and antibiotics are forbidden in aquaculture and in crop agriculture, respectively. Presence and/or accumulation of pesticides in fish, greenhouse atmosphere, and in recirculated water [2,3] could also be problematic. Furthermore, in terms of biological alternative, no biopesticides have been especially developed and registered for aquaponic or hydroponic use. Evaluation and development of microbial biopesticides in aquaponics are currently still at the early stages [1].

Oomycetes pseudo-fungi responsible for root rot diseases, such as *Pythium aphanidermatum* (Edson) Fitzp., are fungal protists able to produce mobile form of dispersion in recirculating water reviewed by [4]. This particularity makes them problematic because of their fast spread in the system and the scarcity of available methods to remove them in aquaponics [1]. Etiology and epidemiology of *Pythium* species in hydroponics were extensively reviewed by Sutton et al. (2006) [4]. From this review, the following key elements could be exposed. *Pythium* diseases especially affect root zone and reduce plant yields and quality. First stages of the infection in root are normally biotrophic and asymptomatic. After these first stages of root colonization, *Pythium* spp. becomes necrotrophic and then induces symptoms. In general, symptoms translate into root discoloration turning in various shades of brown and finally degenerating in decaying and rotting roots. The foliage generally stays asymptomatic, with no perturbation of photosynthesis, for example, until severe root symptoms appear and produce leaf wilting as secondary symptoms. This lack of foliar symptoms makes the disease difficult to diagnose at early stages without inspecting the root zone. Furthermore, some *Pythium* spp. can remain asymptomatic until stressing conditions appear. High temperatures (from 23 °C to 35 °C, depending on the species) in the aerial zone or in the nutrient solution are one of the main factors encouraging fungal growth, while the plant is also directly affected by the stressing conditions of the high temperatures and the resulting decrease of dissolved oxygen in the nutrient solution.

Nevertheless, aquaponic systems could be more outfitted against plant pathogens than first expected. In fact, two recent studies [5,6] and a recent review [1] reported the potentially suppressive (i.e., antagonistic) action of fish effluents or aquaponic water against plant pathogens by the natural presence of beneficial compounds and/or microorganisms. Suppressiveness in soilless culture has already been defined by Postma et al. (2008) [7] as “referred to the cases where (i) the pathogen does not establish or persist; or (ii) establishes but causes little or no damage”. In aquaponic systems, in which water is recirculated, the presence of beneficial microorganisms and organic compounds are suspected to be the key sources of this suppressive action [1]. Amongst beneficial microorganisms, antagonistic microorganisms are the ones suspected to act in suppressiveness against plant pathogens. In a more general way, modes of action of antagonistic microorganisms are commonly classified in: competition for nutrients and niches, parasitism, antibiosis, and/or plants defenses elicitation [8,9,10]. Concerning organic matter, its role in aquaponic suppressiveness could be related to the promotion of plant beneficial microorganisms and/or in plant biostimulation [1]. In the review entitled “Microbial suppressiveness of Pythium damping-off diseases”, Kilany et al. (2015) [11] also supported the importance of organic matter to control *Pythium* diseases. More generally, organic matter or amendments are known to be important factors for diseases suppressiveness [12,13].

Consequently, this study aimed at: (i) evaluating aquaponic water suppressiveness in vitro and for the first time in vivo on lettuce against *P. aphanidermatum*; (ii) differentiating the origin (microorganisms or dissolved compounds) of the in vitro suppressive action; (iii) analyzing and comparing aquaponic water microbiota with hydroponic and complemented (in nutrient salts) aquaponic water in the in vivo test through 16S rDNA and ITS Illumina sequencing; and (iv) identifying which specific microorganisms may be correlated with aquaponic water suppressiveness in the in vivo test.

## 2. Materials and Methods

### 2.1. In Vitro Tests

In vitro tests aimed at evaluating the effect of aquaponic (AP) water recirculated aquaculture system (RAS) water and biofilter media (BM) microorganisms’ suspension on the growth of *P. aphanidermatum* in a V8 CaCO3 broth (see Section 2.1.1). Origin and composition of these waters are detailed in Supplementary Materials S1. Briefly, waters were sampled in the RAS and AP system of Gembloux Agro-Bio Tech, University of Liege, in Belgium. BM microorganisms were recovered by washing biofilter media with 0.05 M Kalium Phosphate Buffer plus 0.05% Tween 80 (KPBT). These 3 types of water were also tested after a 0.2 µm filtration to remove microorganisms.

#### 2.1.1. Methodology

Centrifuge tubes of 50 mL were inoculated with 5 mm plugs of 3-day-old culture of *P. aphanidermatum* (CBS 132490) grown in PDA (Potatoes Dextrose Agar) Petri dishes at 25 °C in the dark. These 50 mL tubes contained 20 mL of clarified V8 CaCO_3_ broth with different compositions based on the modality (Table 1). Two kinds of clarified and autoclaved V8 CaCO_3_ broth were used. The first was a classical V8 CaCO_3_ clarified broth (800 mL of distilled water, 200 mL of V8 juice, and 3 g of CaCO_3_), and the second was a V8 CaCO_3_ broth containing only 75% of its content in distilled water (V8-75% is composed of 550 mL of distilled water, 200 mL of V8 juice, and 3 g of CaCO3). RAS or AP test consisted in 10 centrifuge tubes with 15 mL of V8-75% plus 5 mL of RAS or AP water, respectively, 10 other tubes of 15 mL of V8-75%, plus 5 mL of 0.2 µm filtrated RAS (F-RAS) or AP (F-AP) water to remove microorganism, and 10 last tubes of 20 mL classical V8 CaCO_3_ to serve as positive control for the growth of *P. aphanidermatum*. Lastly, the BM test was conducted by using 10 tubes with 15 mL of V8-75% broth plus 5ml of BM water (obtained through washing of biofilter media in KPBT buffer; see Supplementary Materials S1 for further details) and the positive control constituted in 10 tubes with 15 mL of V8-75%, plus 5 mL of KPBT. These 3 tests are summarized in Table 1. After the broths’ inoculation with the mycelial plugs, tubes were incubated at 25 °C in the dark for 5 days. The mycelium bulks thus produced were weighed after filtration and dried by centrifugation through a cheese cloth at 2350 g during 10 min. Three repetitions were carried out for each test (RAS, AP, and BM) over the course of 3 days, with aliquots of the same water sample kept at 4 °C. These repeated tests were also replicated twice with new water samples taken within one week of interval.

#### 2.1.2. Statistical Analysis

Statistical analyses were performed on Minitab v.19 software (Minitab Inc., State College, PA, USA). Assumptions of normality and homogeneity of variance were checked by Ryan-Joiner and Levene’s tests. The significance of each modality (see Table 1) on *P. aphanidermatum* mycelial growth for the 3 tests (RAS, AP, and BM) was determined by a partially hierarchized 3-way analysis of variance (ANOVA). The factors used are the modality, the repetition (3 repetitions for the same water sample) and the replication (3 replications with new water sample collected within one week of interval). The repetition factor was hierarchized to the replication factor. In case of interaction between factors, the 3-way ANOVA was decomposed in 2- or 1-way ANOVA. Dunnett’s Multiple Comparison test was then used as a post hoc test to compare modalities means to the positive control.

### 2.2. In Vivo Tests

The in vivo test consisted of *P. aphanidermatum* inoculation on lettuce growing with specific environmental condition, in small raft boxes (description in Section 2.2.1.) containing aquaponic (AP) water, complemented aquaponic (CAP) water, and hydroponic (HP) water as treatment. Origin and composition of these waters are detailed in Supplementary Materials S1 and in Table S1. This test was replicated once in time with water sampled in two different dates (trial 1 and 2 in Table S1). For each treatment (i.e., type of water), one raft box containing 4 lettuce plants was used as healthy control (HC modality, i.e., non-inoculated box), and the other one containing also 4 lettuce plants was inoculated by *P. aphanidermatum* (IL modality). Suppressiveness evaluation of AP water was made by suppressiveness indexes comparison with the other HP or CAP treatments. These indexes took into account the HC results-specific of each treatment (see Section 2.2.4 for suppressiveness indexes definition). Composition and diversity of lettuce root microbiota of the first replicate were also analyzed through 16S rDNA and ITS Illumina sequencing analysis (see Section 2.2.5).

#### 2.2.1. Lettuce Cultivation

Organic pelleted lettuce (Lactuca sativa) var. Millennia RZ (Rijk Zwaan, Merksem, Belgium) were sown in 36 × 36 × 40 mm rockwool cubes (Grodan B.V., Roermond, Holland) and placed in a phytotron (Fitotron^®^ SGC 120 Plant Growth Chamber, Weiss Technik, Liedekerke, Belgium) with a day/night photoperiod of 16 h/8 h, a temperature of 22 °C/18 °C (16 h/8 h), and a relative humidity of 65%. Plugs were first placed in round plant trays with 2 cm of tap water over 11 days for the germination stage. The lighting system consisted in two 40-W LED panel of 120 × 30 cm, 6500 K (Novaled GmbH, Dresde, Germany) with specific wavelength spectrum designed for lettuce, and a photosynthetically active radiation (PAR) of 180 µmol.m^−2^·s^−1^. After this germination period, plugs were transplanted into homemade hydroponic boxes reproducing hydroponic raft system (deep water culture). These raft hydroponic boxes were composed of 30L Allibert Crownest boxes (Curver Benelux B.V., Rijen, Holland) of 36.3 × 42.5 × 26.3 cm (L × W × H) with a raft panel cut at box dimension in a rigid extruded polystyrene panel 3 cm thick. In this raft, 4 round holes were drilled in the 4 corners to welcome 5-cm rockwool baskets. The 6 hydroponic boxes were each filled with 20 L of the different water treatments and oxygenated 3 times a day for 15 min by 6 diffuser discs of 10 cm (Hi Oxygen disc, Aquatic Science, Herstal, Belgium) placed at bottom of each box and connected to a 40-W air pump (Hi-Blow 40, Aquatic Science, Herstal, Belgium) set at mid-air flow. These 6 boxes were placed in a shelf into the same Phytotron as for germination, 3 boxes on each floor. The same lighting panels were also used (one by floor) 19 cm over the top of the boxes for 16 h per day. After germination, lettuce plants were grown during 31 days in specific environmental conditions mimicking stressing condition in greenhouse and suitable to *P. aphanidermatum* disease development. During the first 21 days, the phytotron was set at 28/25 °C (d/n; 16 h/8 h) for the temperature and at 65% for humidity. For the last 10 days, the temperature was set at 35/25 °C (d/n; 16 h/8 h) and the humidity at 92%.

#### 2.2.2. Composition, Formulation, and Management of AP, CAP, and HP Waters

Composition of AP, CAP, and HP waters are described in Table S1 in Supplementary Materials, for both trials. HP water is a nutrient solution composed of high purity mineral salts in demineralized water to reach the nutrients concentration recommended in Resh [14] for hydroponic lettuce nutrient solution. The first week after transplantation of the seedlings in the boxes, only one half of salts quantity were added to the nutrient solution (½ HP) in order to avoid osmotic stress. The microorganisms’ concentration at the beginning was determined by the inoculation and plating of 100 µl of ½ HP water on solid PDA (Potatoes Dextrose Agar) and LB (Luria-Bertani) Petri dishes. Number of Colony Forming Unit (CFU) was counted after 3 days of incubation at 23 °C with 18 h/6 h lighting. After this first week of lettuce adaptation, the rest of the salts were added to reach normal Resh nutrients concentration. AP water was characterized to determine its composition in macro and micronutrients, its Biological Oxygen Demand in 5 days (BOD5) and its concentration in cultivable microorganisms. After filtration at 0.45 µm, the concentration in NO^3−^-N, NH^+^-N, PO_4_^3−^-P, K^+^, Ca^2+^, Mg^2+^, SO^4−^-S, and Fe^2+^ was determined using a multiparameter spectrophotometer (HI 83200, HANNA instruments, Woonsocket, RI, USA) with the following reagents: HI 93,700 (TAN), HI 93,728 (NO^3−^), HI 93,717 (PO_4_^3−^), HI 93,751 (SO_4_^2−^), HI 93,750 (K^+^), HI 93,752 (Ca^2+^), and HI 93,752 (Mg^2+^). BOD5 was measured by OxiTop^®^ (WTW Gmbh and Co, Welheim, Germany) manometer method following the standard method ISO 16072:2002. Microorganisms concentration was calculated as already described for HP water. Regarding CAP water, osmotic stress was also avoided the first week after transplantation by using the same classical AP water as described before but with a pH adjusted to 5.5–5.8. Thereafter, high purity nutrients salts were added to the solution to reach nutrients concentration levels of HP Resh nutrients solution. Salts used for HP and CAP were the following: MgSO_4_·7H_2_O, Mg(NO_3_)_2_.6H_2_O, NH_4_NO_3_, K_2_HPO_4_, Ca(NO_3_)_2_·4H_2_O, KNO_3_, K_2_SO_4_, Fe-EDTA, MnSO_4_·4H_2_O, CuSO_4_·5H_2_O, ZnSO_4_·7H_2_O, (NH_4_)6Mo_7_O_24_·4H_2_O, and H_3_BO_3_. Calculations of salts quantity needed for ½ HP, HP, and CAP waters were performed on HydroBuddy free software (http://scienceinhydroponics.com/category/hydrobuddy). For each type of water, pH and electroconductivity (Ec) were measured 3 times a week, and pH was adjusted to the right level with H_2_SO_4_ 1M or NaOH 1M. For HP, AP, and CAP solutions, the pH was, respectively, kept between 5.5–5.8, 7.0–7.5, and 5.5–5.8. These parameters were measured with a multimeter (model HQ40d, HACH, Loveland, CO, USA) equipped with 2 probes (pH and Ec).

#### 2.2.3. Lettuce Inoculation by *P. aphanidermatum*

*Pythium aphanidermatum* (CBS 132490) was grown in PDA Petri dishes at 23 °C with 18 h/6 h lighting for 3 days. Sterile 150 mL Erlenmeyer flasks containing 25 mL of clarified V8 CaCO_3_ broth (800 mL of distilled water, 200 mL of V8 juice, and 3 g of CaCO_3_) were then inoculated by 5-mm plugs of the *P. aphanidermatum* culture. The Erlenmeyer was closed with a cotton ball and incubated during 6 days at 23 °C with 18 h/6 h lighting. The mycelial bulk thus produced was recovered and rinsed by vortexing in a 50 mL centrifuge tube filled with 15 mL of sterile isotonic water (0.85% NaCl). The operation was repeated minimum twice until V8 color loss. Then the mycelium was drained on a sterile paper towel and mixed 8 times during 3 s with a hand blender (Braun Minipimer Control Plus, 300w) in a sterile solution containing 10 mM of sucrose and 0.05% of Tween 20 in distilled water. The proportion used was 5 mg of mycelium for 1ml of solution. The resulting propagules suspension corresponds to a mean concentration of 5.33 × 10^3^ propagules/mL. Ten ml of this suspension were inoculated per rock-wool plug after 5 and 12 days (after seedlings transplantation) of lettuce growth. The 3 water treatments with the lettuce plants inoculated with *P. aphanidermatum* were then AP-Pa, HP-Pa, and CAP-Pa. For the healthy lettuce, 10 mL of sucrose plus tween solution was added per rock wool plug in the healthy controls (HC) boxes.

#### 2.2.4. Suppressiveness Measures

On the last day of the experiment, rating of root rot symptoms were recorded and lettuce plants were harvested to weigh fresh foliar mass. Leaves were then dried in a laboratory oven at 70 °C during 48 h and weighed. Root rot was recorded according to the following scale (adapted from Reference [15]):

0 = 100% of healthy white roots, no discoloration;
1 = less than 50% of healthy light brown roots or white roots with brown apex;
2 = more than 50% of healthy light brown roots or white roots with brown apex;
3 = less than 50% of unhealthy medium brown roots with a possible decaying part;
4 = more than 50% of unhealthy medium brown roots with a possible decaying part;
5 = less than 50% of brown-black decaying or dead roots;
6 = more than 50% of brown-black decaying or dead roots.

To be able to compare water suppressiveness of each treatment on *P. aphanidermatum* independently of their respective performance without the disease, different indexes were calculated. Relative foliar turgidity decrease (FTD) represents the relative decrease in leaf water content of *P. aphanidermatum* inoculated lettuce (IL) compared to the water content mean of the corresponding healthy control (HC). FTD was calculated as follows:

(1) $$\mathrm{FTD}=100\;\times \;\left({\mathrm{FWC}}_{\mathrm{of}\;\mathrm{HC}\;\mathrm{mean}}-{\mathrm{FWC}}_{\mathrm{of}\;\mathrm{IL}}\right),$$

(2) $$\mathrm{FWC}=\;\frac{F_{f}M-F_{d}M}{F_{f}M},$$

where FTD is the relative foliar turgidity decrease, FWC the foliar water content, F_f_M the foliar fresh mass, F_d_M the foliar dry mass, HC the healthy control, and IL the inoculated lettuce.

Foliar fresh mass decrease (F_f_MD) was also calculated by comparison of IL foliar fresh mass with the mean of the corresponding HC foliar fresh mass. The equation is the following:

(3) $$F_{f}\mathrm{MD}=100\;\times \frac{F_{f}M_{\;\mathrm{of}\;\mathrm{HC}\;\mathrm{mean}}-F_{f}M_{\;\mathrm{of}\;\mathrm{IL}}}{F_{f}M_{\;\mathrm{of}\;\mathrm{HC}\;\mathrm{mean}}},$$

where F_f_MD is the foliar fresh mass decrease, and F_f_M is the foliar fresh mass.

Foliar dry mass decrease (F_d_MD) was calculated by comparison of IL foliar dry mass with the mean of the corresponding HC foliar dry mass. The equation is the following:

(4) $$F_{d}\mathrm{MD}=100\;\times \frac{F_{d}M_{\;\mathrm{of}\;\mathrm{HC}\;\mathrm{mean}}-F_{d}M_{\;\mathrm{of}\;\mathrm{IL}}}{F_{d}M_{\;\mathrm{of}\;\mathrm{HC}\;\mathrm{mean}}},$$

where F_d_MD is the foliar dry mass decrease, and F_d_M is the foliar dry mass.

Lastly, a corrected root rot rating (CRRR) was calculated by taking into account the score of the corresponding HC.

(5) $$\mathrm{CRRR}={\;\mathrm{RRR}}_{\;\mathrm{of}\;\mathrm{IL}}-{\mathrm{RRR}}_{\;\mathrm{of}\;\mathrm{HC}\;\mathrm{mean}},$$

where CRRR is the corrected root rot rating, and RRR is the root rot rating.

#### 2.2.5. Statistical Analysis of Suppressiveness Indexes

Statistical analyses were performed on Minitab v.19 software (Minitab Inc., State College, PA, USA). Assumptions of normality and homogeneity of variance were checked by Ryan-Joiner and Levene’s tests. The significance of each kind of water (HP, AP, and CAP) on relative foliar turgidity decrease (FTD), foliar fresh mass decrease (F_f_MD), foliar dry mass decrease (F_d_MD), and corrected root rot rating (CRRR) was determined by a 2-ways analysis of variance (ANOVA). The factors used were the type of water and the replication. In case of interaction between factors, the 2-way ANOVA was decomposed in 1-way ANOVA. Tukey Multiple Comparison test was used as a post hoc test to pairwise compare types of water.

#### 2.2.6. Microbiota Analysis of the First Test

##### Microbiota Sampling

The microbial communities from 3 lettuce root compartments, the rhizosphere, rhizoplane, and endosphere, were sampled on the last day of the first in vivo experiment. In this experimental setup, the rhizosphere is the water area directly influenced by the roots. Consequently, in this experiment, the rhizosphere corresponds to the water in the boxes. Therefore, one water sample of 30 mL per box was taken, mixed with 10 mL of autoclaved glycerol and then immediately frozen in liquid nitrogen and stored at −20 °C. The rhizoplane is the roots surface including particles and microorganisms adhering on it. The rhizoplane microbiota was recovered as follows [16]: 0.5 g of roots of each of the 4 lettuce plants were individually sampled and sonicated separately in 30 mL of KPBT (pH 6.5) during 10 min. Roots were removed from the 50 mL centrifuge tubes and 10 mL of autoclaved glycerol was added to the buffer containing the microbiota before flash freezing in liquid nitrogen and conservation at −20 °C. Roots used for the rhizoplane collection were then disinfected and washed for endospheric microbiota analysis. Disinfection was achieved by immerging the roots of each lettuce in alcohol (99%) for 1 min, then in sodium hypochlorite (3.78%) for 3 min, and then rinsed 3 times in sterile distilled water during 3 min. Disinfected roots were then flash frozen separately in liquid nitrogen and stored at −80 °C for further analysis.

##### Samples Preparation and DNA Extraction

All samples were processed under sterile conditions before DNA extraction. Rhizoplane and rhizosphere samples were defrosted and filtered through sterile cheesecloths to remove root residues. The filtrates were then vacuum filtered through sterile 0.2 µm filter (47 mm Supor^®^ 200 PES Membrane Disc Filter, PALL Corporation, Portsmouth, UK). The filters were cut in small pieces and temporary stored at 4 °C before DNA extraction on the same day. Defrosted root samples (i.e., for the endosphere analysis) were grinded in mesh bags (12 × 12.5 cm, Agdia Biofords, Elkhart, IN, USA) containing KPBT buffer with the root:buffer ratio of 1:9. Root tissues inside the bags were grinded with a smooth disk tip mounted on a drill (model 850 W PowerPlus X0270, Varo, Lier, Belgium). Resulting root saps were recovered and filtered through sterile cheesecloths before being flash frozen and conserved at −20 °C with 25% autoclaved glycerol. After defrosting, root saps were concentrated through centrifugation at 2350× *g* for 20 min at 20 °C. Supernatant were removed and the concentrated part (1/4 of the volume) was used for DNA extraction.

FastDNA Spin Kit using Cell Lysis Solution TC (MP Biomedicals, Illkirch-Graffenstaden, France) was used according to manufacturer’s instructions to extract DNA microbiota from filters for rhizosphere and rhizoplane samples and from concentrated sap for endosphere samples. DNA quality was checked with a Nanodrop (Nanodrop ND-1000 Spectrophotometer, Nanodrop Technologies, Wilmington, DE, USA) and then stored at −20 °C before amplification.

##### Amplification and Sequencing

DNA amplification was performed with the 2X KAPA HiFi HotStart ReadyMix PCR kit (Kapa Biosystems) according to manufacturer’s instructions. For bacterial community analyses, composite primers used for 16S rDNA amplification of V1–V3 hypervariable regions were the Forward 27F and Reverse 534R with Illumina sequencing adapters in 5′ [16,17]. For fungal community analyses, the ITS1 DNA region was targeted with the primers ITS1-F_ KYO2 and ITS2_KYO2 [18], with the same sequencing adapter in 5′. Amplifications were carried out on thermocycler with an initial denaturation step at 95 °C for 5 min followed by 25 (for all 16S rDNA samples), 30 (for ITS rhizosphere and ITS rhizoplane samples), or 35 cycles (for ITS endosphere samples) of denaturation at 95 °C for 20 sec, annealing at 55 °C for 30 s and elongation at 72 °C for 30 s. A final elongation step was performed at 72 °C for 5 min [16]. The PCR products were further tagged and sequenced by paired-ends Illumina MiSeq at DNAVision (Gosselies, Belgium) with a run of 250 nucleotides.

##### Bioinformatics and Statistical Analyses

Demultiplexed data obtained from DNAVision were imported in the QIIME 2 software (q2) version 2019-4 [19] as single-end fastq files with forward reads only for 16S and paired-end fastq files for ITS. Sequences used are available on SRA-NCBI platform (https://www.ncbi.nlm.nih.gov/sra) with BioProject ID PRJNA662206. The workflow used was similar to [16]. Briefly, q2 VSEARCH feature-classifier plugin was used after quality control with DADA2 method. Reference database SILVA_132 release for 16S rDNA version 10.04.2018 and UNITE release for fungi version 18.11.2018 were used at 99% of sequence similarity. Q2 taxa filter-table script was run to discard cytoplasmic contaminations. Rhizosphere, rhizoplane and endosphere (i.e., type of microbiota) samples were separated with q2 feature-table script. Alpha and beta diversities were calculated using the q2-diversity core-metrics-phylogenetic plug-in with microbiota specific rarefaction. Rarefaction levels were chosen to keep the maximum of sequences by sample provided that a plateau is reached in the alpha rarefaction curves previously generated. Alpha diversity indexes (Observed OTUs number and Shannon index) were compared with the Kruskal-Wallis pairwise test. Beta diversity index (Weighted Unifrac distance metrics) were compared by the pairwise PERMANOVA (999 permutations) pseudo-F test. DS-FDR (Discrete False-Discovery Rate) tests with Kruskal–Wallis controlling procedure were carried out to compare OTUs relative abundance between treatments microbiotia. Rhizosphere samples were composed of a unique liquid sample per treatment thus preventing statistical analysis of its microbiota. Linear correlations and ANOVA were done in R statistical software version 3.6.0 between α-diversity indexes of rhizoplane HC of HP, AP, and CAP water, and suppressiveness indexes (FTD, FfMD, FdMD, and CRRR). Relationships between OTUs relative abundances at species level of rhizoplane HC of HP, AP, and CAP water, and suppressiveness indexes (FTD, FfMD, FdMD, and CRRR) were also tested in R. For these correlations, only the rhizoplane microbiota was selected in accordance to β-diversity results.

## 3. Results

### 3.1. In Vitro Test

**Figure 1:**
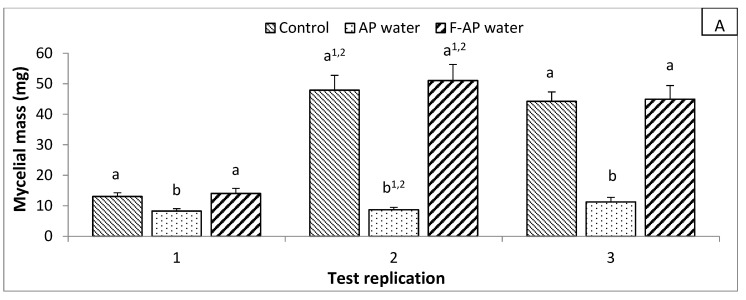

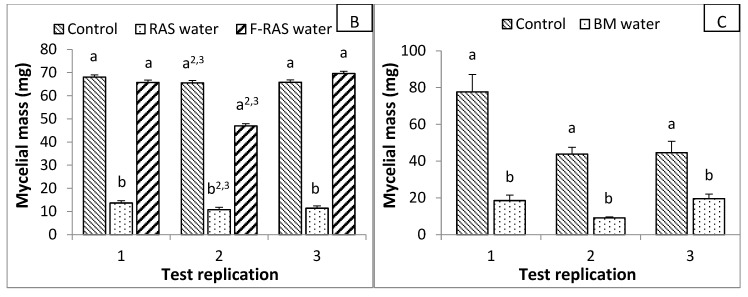
*P. aphanidermatum* mycelial growth when testing (**A**): AP and F-AP water; (**B**) RAS and F-RAS water; and (**C**) BM water. Bars indicate the standard error of the mean. Different letters indicate significant differences by Dunnett’s ANOVA post hoc test (*p* ≤ 0.05) between modalities inside a same test replication, i.e., within the same date of water collection. Exponents in these letters indicate that the significance is valid only for the repetition (1, 2, or 3) mentioned by the numbers.

For each water treatment (AP, RAS, and BM waters), statistics showed significant interactions (3-way ANOVA; *p* ≤ 0.05) between factors (the modality, the replication, and the repetition), then inhibiting effect of the modality was tested for each replication independently (i.e., week replication 1, 2, or 3 done with different water samples) and represented in Figure 1. An inhibiting effect of AP, RAS and BM waters on *P. aphanidermatum* mycelial growth was observed when used without previous 0.2µm filtration step (Figure 1). Filtrated waters did not differ from the control. Although this observation was significant (2-way ANOVA; *p* ≤ 0.05) for nearly all tests repetitions inside a week replication, some exceptions could be noticed after ANOVA separation, depending on the repetition factor (i.e., repetition 1, 2, or 3) and, thus, in case of interactions between the “modality” and “repetition” factors.

AP water had a significant inhibiting effect on mycelial growth during week replication 1 (−36.6%; *p* = 0.005) and 3 (−74.6% *p* = 0.000) by 2-way ANOVA, while F-RAS has no significant effect compared to the control. For the replication week 2, the significant effect of AP water was observed for the repetition 1 and 2 (*p* = 0.000 for both) but not for week 3 (*p* = 0.098) by 1-way ANOVA.

RAS water had a significant (2-way ANOVA; *p* = 0.000) inhibiting effect on mycelial growth during week replication 1 (−79.9%) and 3 (−82.7%), while F-RAS had no significant effect. In week 2, this significant effect of RAS water was also observed but only for the repetition 2 (1-way ANOVA; *p* = 0.000) and 3 (1-way ANOVA; *p* = 0.000).

BM water significantly (2-way ANOVA; *p* = 0.000) decreased mycelial growth of *P. aphanidermatum* for all 3-week replications compared to the control (−76.1%, −79.3%, and −56.2%, respectively). But it should be noted that the statistical repetition factor had a significant effect by 2-way ANOVA for the replication factor week 1 (*p* = 0.023) and 3 (*p* = 0.000).

### 3.2. In Vivo Test

#### 3.2.1. Suppressiveness

**Figure 2:**
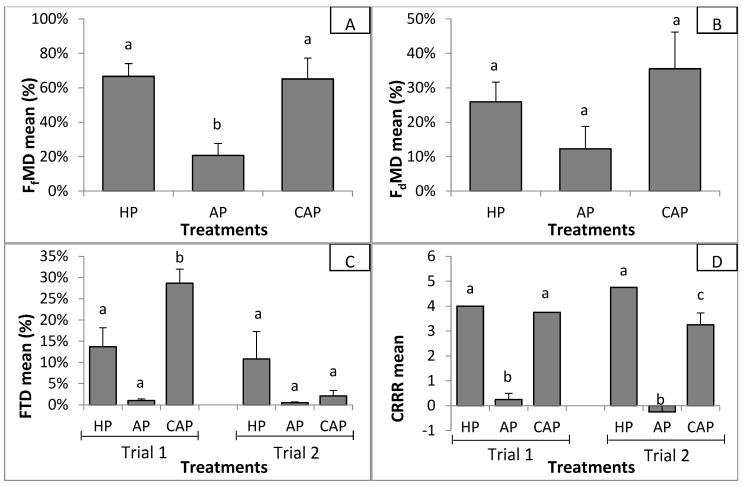
Effect of hydroponic HP, AP, and complemented aquaponic CAP treatment on (**A**) foliar fresh mass decrease (FfMD), (**B**) foliar dry mass decrease (FdMD), (**C**) relative foliar turgidity decrease (FTD), and (**D**) corrected root rot rating (CRRR) suppressiveness indexes. Bars indicate the standard error of the mean. Different letters indicate significant differences between treatments by Tukey’s ANOVA post hoc test (*p* ≤ 0.05).

Results indicated that AP lettuce stayed healthier than HP and CAP lettuce in the presence of the pathogen for the 4 suppressiveness indexes considered (Figure 2).

Statistical analyses indicated significant effect of the replication on F_f_MD and F_d_MD (2-way ANOVA; *p* = 0.001 and *p* = 0.006, respectively) that were lower during the second trial. Concerning the effect of the treatment (HP, AP, or CAP water), F_f_MD was significantly lower (2-way ANOVA; *p* = 0.000) when AP water was used compared to HP and CAP water (Figure 2A). Means were 20.7% for AP, 66.6% for HP, and 65.1% for CAP. A similar trend (i.e., 12.3%, 25.9%, and 35.3% for AP, HP, and CAP water, respectively) was also observed for F_d_MD but was not significant (Figure 2B). Interaction between the test replication (trial 1 or 2) and the treatment was recorded by 2-way ANOVA for FTD (*p* = 0.003) and CRRR (*p* = 0.028) suppressiveness indexes. Effects of the treatment were then analyzed separately depending of the replication, as illustrated in Figure 2C,D. Despite the fact that FTD of CAP was 28.7%, HP was 13.7%, and AP was 0.98% for the first trial, the Tukey’s 1-way ANOVA post hoc test only highlights a significant FTD difference (*p* = 0.001) of CAP water treatment compared to the two others waters (which show no difference). During the second trial, no treatment difference was calculated by 1-way ANOVA for FTD but means tended to show a lower FTD for AP water. FTD were 2.1% for CAP water, 0.5% for AP water, and 10.8% for HP water. In regard to the CRRR index of the first trial, CRRR in AP (i.e., 0.25) was significantly lower (1-way ANOVA; *p* = 0.000) than in HP (i.e., 4.00) and CAP (i.e., 3.75) treatments. For the second trial, means of CRRR were −0.25 for AP, 4.75 for HP, and 3.25 for CAP. All treatments were different (1-way ANOVA; *p* = 0.000) between them.

#### 3.2.2. Microbiota Composition and Diversity

Several samples were removed throughout the analysis. For the analysis of rhizoplane ITS community, 3 out of 4 CAP samples were not sequenced because no band was observed on the electrophoresis gel after PCR amplification. Another source of removal was the generation of a too low number of sequences. Consequently, 2 samples out of 4 were removed for AP, AP-Pa, and HP treatments in the 16S rDNA endosphere analysis. The last remaining CAP sample for ITS rhizoplane analysis was also removed for lack of sequences.

##### Microbiota Composition

Bar charts with 16S rDNA and ITS relative composition, at family level, are presented in Supplementary Materials S2 (Figures S1–S7).

The predominant family represented for bacteria was mainly the Bulkolderiaceae. This family was present in all treatments with a minimum relative abundance of 39.7%, 19.0%, and 12.7% in the endosphere, rhizoplane, and rhizosphere, respectively. However, in the rhizosphere, Methylophilaceae was present at higher abundance (17.6% at minimum), except in AP treatment (7.2%). In the endosphere, it is interesting to note that the Pseudomonadaceae family was important in all HC (8.7% at minimum) but that this ratio decreased when *P. aphanidermatum* was present. In other root microbiota, the Pseudomonadaceae family was lower than 1% of relative abundance. In the rhizoplane, Sphingomonadaceae and Lactobacilliaceae were also relatively abundant and more, especially, in AP, with a mean of 9.9% and 6.1%, respectively. The Xanthomonadaceae were also predominant in the rhizoplane but not in non-inoculated AP treatment.

Concerning ITS, most of the taxa were unassigned and/or only classified down to the *Fungi* kingdom. The part of unassigned sequences represented 11.59%, 52.9%, and 66.6% in average of the total OTUs number in endosphere, rhizoplane, and rhizosphere, respectively. However, a second analysis using a eukaryote UNITE database (results not showed) indicated that up to 5.5% of unassigned OTUs could be assigned to the Stramenipiles, Protista, and Viridiplantae kingdoms. Furthermore, manual blast on the NCBI platform (https://blast.ncbi.nlm.nih.gov/Blast.cgi) allowed the identification of some abundant OTUs to the Prostista kingdom (see the ITS specific case in Section 3.2.3., where *Protista* represented an abundance of 11.74% on 52.44% of unassigned OTUs). Beyond that, Aspergilaceae were well represented (between 3.2% and 15.6%) in endosphere, as well as the Pleosporales, Ustilaginales, and Dothideales orders. In the rhizoplane, the Debaryomycetaceae was the most assigned family in all treatments at a minimum of 13.2%. The second most represented assigned family was Catenariaceae (4.1% at minimum) but not in HP and HP-Pa treatments, where it was lower than 1.7%. In the rhizosphere, AP water was dominated by Pleosporales, Ustilaginales, and Dothideales orders and, to a lesser extent, other treatments.

##### Microbiota α-Diversity

Global views of α-diversity indexes (observed_OTU number and Shannon index) for the 3-root microbiota are shown in Figures S7 and S8 in Supplementary Materials.

##### a. Endosphere

**Figure 3:**
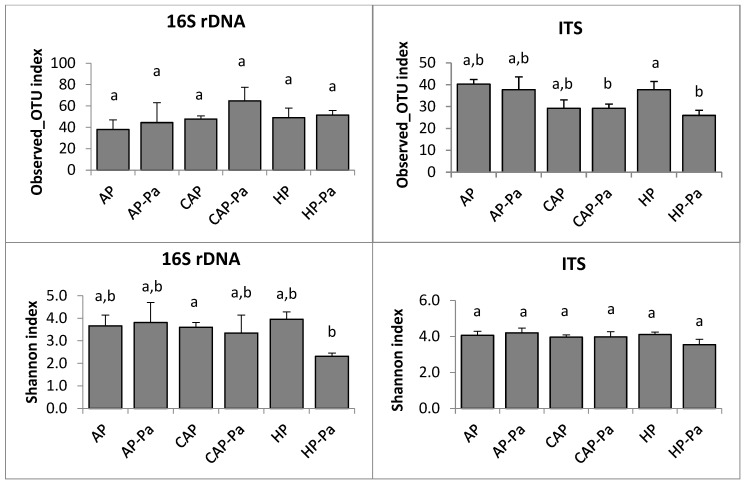
Species richness (observed_OTU number) and species diversity (Shannon index) of lettuce endosphere of 16S rDNA and ITS analyses depending on the treatment (AP, AP-Pa, CAP, CAP-Pa, HP, and HP-Pa). Bars indicate the standard error of the mean. Treatments that do not share a same letter are significantly different by Kruskal-Wallis pairwise test (*p* ≤ 0.05).

Species richness (observed_OTU number) and species diversity (Shannon index) of lettuce endosphere were shown in Figure 3. Endosphere species richness (observed_OTU number) was relatively similar to all treatments. No statistical differences were found in bacterial analysis, while some pairwise differences (*p* ≤ 0.05) were observed in ITS analysis by Kruskal-Wallis test. As for the richness, the species diversity (Shannon index) of the endosphere was relatively similar, whatever the treatment. Sole HP-Pa Shannon index was significantly different from CAP Shannon index (Kruskal-Wallis; *p* = 0.02) and only in the 16S analysis.

##### b. Rhizoplane

**Figure 4:**
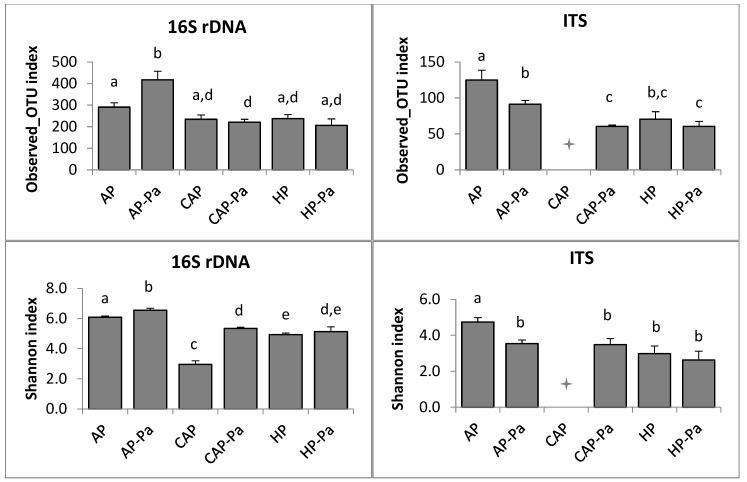
Species richness (observed OTU number) and species diversity (Shannon index) of lettuce rhizoplane of 16S rDNA and ITS analyses depending on the treatment (AP, AP-Pa, CAP, CAP-Pa, HP, and HP-PA). Bars indicate the standard error of the mean. Treatments that do not share a same letter are significantly different by Kruskal-Wallis pairwise test (*p* ≤ 0.05). 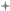 CAP treatment in the ITS rhizoplane was removed by the rarefaction process during bioinformatic analysis.

Concerning lettuce rhizoplane, species richness (observed OTU number) and species diversity (Shannon index) are shown in Figure 4. Richness of AP-Pa was higher (Kruskal-Wallis; *p* ≤ 0.05) than other treatments for bacteria. In ITS, both AP and AP-Pa were higher (Kruskal-Wallis; *p* ≤ 0.05). For 16S species diversity, AP and AP-Pa Shannon indexes were significantly higher (Kruskal-Wallis; *p* ≤ 0.05) than all other treatments. More especially, CAP species diversity was significantly reduced compared to AP (Kruskal-Wallis; *p* = 0.021), indicating that the modification of nutrient elements concentrations and pH of AP water decrease bacterial species diversity. In the ITS analysis, only AP Shannon index was statically different from the others (Kruskal-Wallis; *p* ≤ 0.05) with the highest species diversity. It was also interesting to notice, in the rhizoplane study, that AP-Pa richness and diversity in the 16S analysis were significantly higher from all the others (Kruskal-Wallis; *p* ≤ 0.05), while it was AP in ITS analysis (Kruskal-Wallis; *p* ≤ 0.05).

##### c. Rhizosphere

**Figure 5:**
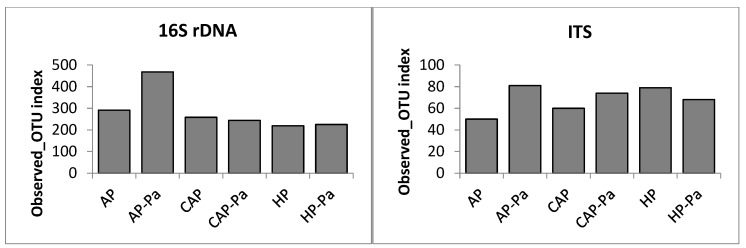

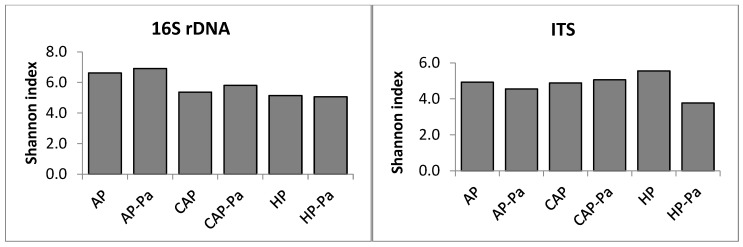
Species richness (observed OTU number) and species diversity (Shannon index) of lettuce rhizosphere of 16S rDNA and ITS analyses depending on the treatment (AP, AP-Pa, CAP, CAP-Pa, HP, and HP-PA). Rhizosphere microbiota was constituted of a unique sample by treatment and was not subject to statistical analysis.

Species richness (observed OTU number) and species diversity (Shannon index) of lettuce rhizosphere were shown in Figure 5. Rhizosphere microbiota was not subject to statistics because of a unique sample per treatment. Consequently, non-statistical interpretations of the rhizosphere showed that AP-Pa had the higher richness number in bacteria followed by AP. In ITS analysis, AP had the lowest. Shannon indexes of AP and AP-Pa seemed higher in 16S rDNA analysis. In ITS, it was HP that showed the higher diversity but that decreased after *P. aphanidermatum* inoculation.

##### Microbiota β-diversity

**Figure 6:**
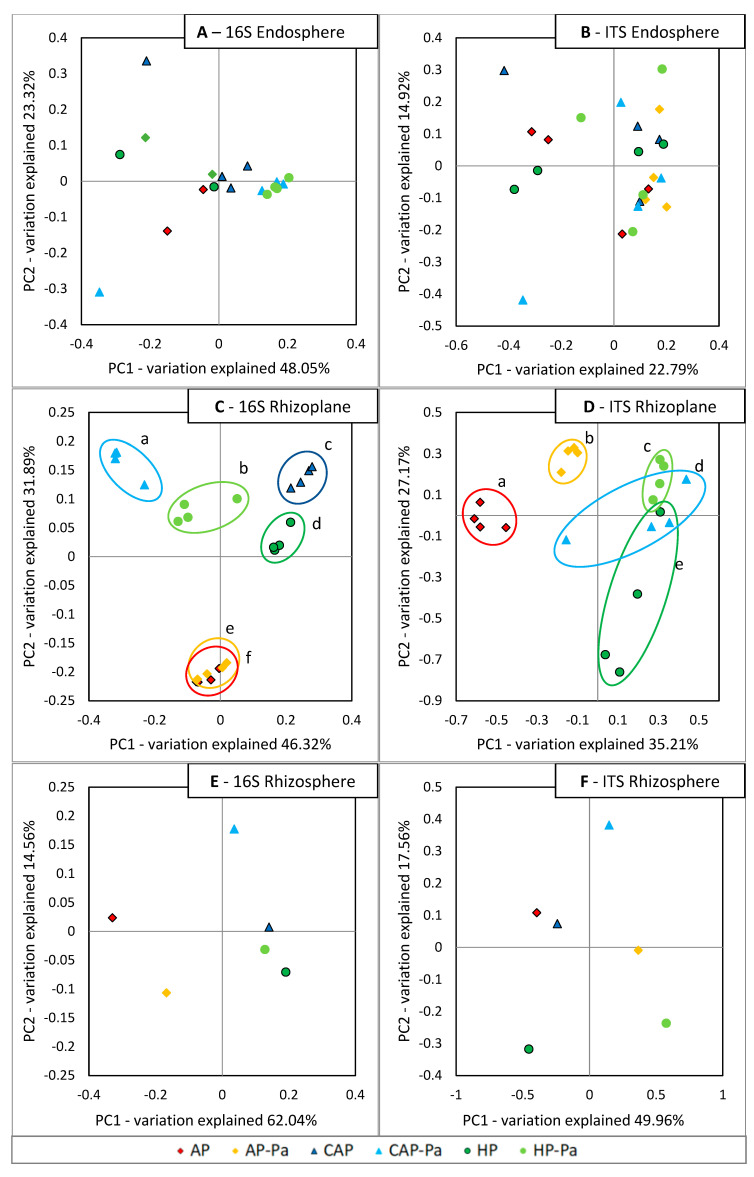
PCoA plots (Principal Coordinates Analysis) of weighted Unifrac distance metrics of 16S rDNA lettuce endosphere (**A**), rhizoplane (**C**), and rhizosphere (**E**) and ITS lettuce endosphere (**B**), rhizoplane (**D**), and rhizosphere (**F**) analyses depending on the treatment (AP, AP-Pa, CAP, CAP-Pa, HP, and HP-PA). In case of differences (*p* ≤ 0.05) with Adonis test, effect of the treatment was pairwise PERMANOVA analyzed, and differences (*p* ≤ 0.05) were indicated by circles with different letters. CAP treatment in the ITS rhizoplane was removed by the rarefaction process during bioinformatic analysis. Rhizosphere microbiota is constituted of a unique sample by treatment and was not subject to statistical analysis.

β-diversity of endosphere, rhizoplane and rhizosphere samples are represented with Principal Coordinates Analysis (PCoA) plots in Figure 6. Statistical differences of β-diversity with Adonis test were observed only in the rhizoplane for both 16S rDNA (*p* = 0.001) and ITS (*p* = 0.001) analyses. Each β-diversity treatment was significantly different (*p* ≤ 0.05) from the other ones according to a PERMANOVA test. However, in 16S rhizoplane, AP and AP-Pa PCoA clusters stayed relatively close to each other (also in PC3 axis, with 0.04 of difference in mean) but still statistically different. AP and AP-Pa clusters were also well separated from the other treatments on the PC2 axis. Modifications of AP water to obtain CAP water (pH drop and salts addition) produced a shift of microorganisms visible in the β-diversity analysis of 16S rhizoplane. For CAP and HP treatments, the addition of *P. aphanidermatum* seemed to induce a translation toward negative value on PC1 in the 16S rhizoplane. In ITS rhizoplane a positive translation on PC2 PCoA axis was visible in HP when *P. aphanidermatum* was present. In comparison with the 16S rhizoplane, *P. aphanidermatum* presence implied a higher β-diversity modification/translation between AP and AP-Pa samples in ITS rhizoplane PCoA. Concerning endosphere, no differences were observed, as well as real trends.

#### 3.2.3. Link between Suppressiveness and Microbiota

Alpha diversity correlation with suppressiveness indexes indicated that a higher richness (observed_OTU number) and diversity (Shannon index) correlated with disease suppression (i.e., lower suppressiveness indexes). This relation was represented by a negative correlation coefficient in Table 2. This relation was significant (ANOVA; *p* ≤ 0.05) for the species diversity essentially and less for the species richness.

Correlations of relative 16S rDNA OTUs abundances with suppressiveness indexes indicated that 92 out of 1018 bacterial OTUs could be linked to suppressiveness. Out of these 92 OTUs, only 18 and 28 OTUs were, respectively, found in HP and CAP but with lower abundance than in AP. Correlations of the top 30 most relative abundant OTUs (representing an abundance of 72.8%) in AP were in most cases significantly correlated (ANOVA; *p* ≤ 0.05) with suppressiveness indexes for 16 rDNA analysis (Table 3). Furthermore, out of these 30 OTUs, only one (f_Methylophilaceae; *g*_*Methylophilus*; s_unculturedbact) was found not to be statistically different in abundance between treatments (AP, CAP, and HP) according to DS-FDR test. The 29 other OTUs were all significantly more abundant in AP compared to HP and CAP treatment (DS-FDR; *p* ≤ 0.05). However, correlations in Table 3 were less significant for F_d_MD index and that could be explained by the fact that no significant differences were found between treatments for this index (see Section 3.2.1). All the values were negative, except for the last one. Negative correlations indicated that, the more abundant the microorganism, the more suppressed the disease was (i.e., lower suppressiveness indexes), and inversely. Moreover, OTUs significantly negatively correlated with suppressiveness indexes (ANOVA; *p* ≤ 0.05) in Table 3 were each time more abundant in AP than CAP or HP. *Methyloversatilis* was the most abundant genus link to suppressiveness followed by Burkholderiaceae family and *Sphingobium* genus. Moreover, these last two taxa were found several times in the top 30. Furthermore, inside Burkholderiaceae, the genus *Hydrogenophaga* was identified twice in the top 30.

Correlations of relative ITS OTUs abundances with suppressiveness indexes indicated that 35 out of 349 fungal OTUs could be linked to suppressiveness. Out of these 35 OTUs, only 11 OTUs were found in HP. Among these OTUs, only one OTU corresponding to *Meyerozyma* genus tended (DS-FDR; *p* = 1) to be more abundant in HP compared to AP (NB: CAP samples were removed from the analysis during rarefaction process). For ITS correlations, in comparison with 16S rDNA, less OTUs were significantly (ANOVA; *p* ≤ 0.05) negatively correlated with suppressiveness indexes in the top 30 most abundant OTUs (representing an abundance of 83.0%) in AP (Table 4). Out of these 30 OTUs, only 14 OTUs were found to be statistically different in abundance (DS-FDR test; *p* ≤ 0.05) between treatments (AP and HP). All these 14 OTUs were found to be more abundant in AP treatment compared to HP treatment. Furthermore, most OTUs were unassigned in QIIME 2 and were thus manually blasted in NCBI platform (https://blast.ncbi.nlm.nih.gov/Blast.cgi) for additional information. This manual blast highlighted that 11.74% of the 52.44% unassigned OTUs of Table 4 were potentially members of the Protista Kingdom. As for 16S rDNA, less significant correlations were found with F_d_MD index. Among identified fungal taxa (for specific OTU) potentially linked to suppressiveness, *Catenaria* genus, Rhizophydiales, and Hypocreales (f_Hypocreales_fam_Incertae_sedis) order could be cited. *Meyerozyma* genus was significantly positively correlated for F_d_MD (ANOVA; *p* = 0.022) and FTD (ANOVA; *p* = 0.017) indexes and tended to be more abundant in HP water, thus indicating that more it was abundant, more the symptoms tended to be high.

## 4. Discussion

Results of in vitro tests indicated that the pathogen was inhibited by microorganisms of AP, RAS and BM waters and not by the mineral or organic compounds found in the waters. However, significance was sometimes influenced by the replication (test replicated with different water samples) or the repetition (test repeated with a same water sample) factor. Interactions between these statistical factors and the mycelial growth were probably due to the growth variability of *P. aphanidermatum* [20]. This assumption is strengthened by differences of growth observed for the controls between repetitions or replications. For example, there was a difference of 72.8% of mycelium mass growth for the control between the replication 1 and 2 in the AP test.

The in vitro effect of fish waters on fungal plant disease can be corroborated by other studies. Indeed, Gravel et al. (2015) [5] reported that aquaculture effluents could promote plant growth, decreased *Pythium ultimum* and *Fusarium oxysporum* growth in vitro and also reduced tomato roots colonization by these fungi. In vitro growth inhibition of *Pythium ultimum* and *Fusarium oxysporum* were much higher when crude fish effluents were used compared to filtered sterilized or autoclave ones. In a paper by Sirakov et al. (2016) [6], bacterial isolates from an aquaponic system showed an inhibitory effect on *P. ultimum* by agar diffusion method.

These in vitro trials informed on the direct effect of AP, RAS, and BM waters against *P. aphanidermatum* in absence of its plant host. However, plant elicitation by microorganisms is a possible antagonistic indirect path to control plant pathogens and cannot be discarded. These results also do not indicate if compounds in solution can help or not control the disease by an indirect way. For example, dissolved organic matter, such as humic acid, met in aquaponics [21,22] cited by [23] can act on plant health by biostimulation or elicitation [24,25,26]. Moreover, plant elicitation by microorganisms or compounds is a possibility of indirect mode of antagonism that cannot be tested in this kind in vitro experiment.

In vivo experiments are better ways to testify the efficiency of antagonistic microorganisms against plant pathogens. In fact, complex interactions between microorganisms, plants, plant pathogens, and the environment are misrepresented in in vitro tests and often give incorrect results when compared to in vivo tests [27]. However, in our case, in vivo tests results confirmed the suppressive action of AP water found in vitro against lettuce *P. aphanidermatum* disease.

Until now, articles studying microbial diversity in AP system are scare. No paper has described fungal composition, while 4 could be cited for bacteria in aquaponic system growing lettuce [16,17,28,29]. The two last references [16,17] originated from the same system than ours. When comparing the results of the 4 references with our study at the family level, *Burkholderiaceae* (including *Comamonadaceae* in UNITE taxonomy database) and Sphingomonadaceae are relatively common in aquaponic lettuce root microbiota. *Comamonadaceae* and *Sphingomonadaceae* were also major taxa in root microbiota of lettuce cultivated in soil [30]. Moreover, according to the list of taxa associated with disease suppressive soils in Expósito et al. (2017) [31], both families or their members are commonly identified and suspected to play a role in suppressive soil. In the lettuce bacterial endosphere of this study, *Pseudomonadaceae* was the second most abundant family after *Burkholderiaceae*. *Pseudomonadaceae*, especially *Pseudomonas* genus, is well known for its antagonistic activities against plant pathogens and was suspected to be an important player in aquaponic suppressiveness [1]. Concerning fungal family found in the root zone in this study, particularly well abundant in the endosphere, the Aspergillaceae family (taxonomy of UNITE database) contains important genera known to be antagonistic fungi and component of suppressive soil. Two genera related to suppressive soil could be cited: *Aspergillus* and *Penicillium* [31]. Supplementary analyses using eukaryote UNITE database and manual blast on NCBI highlighted that a relatively important amount of ITS OTUs could belong to other taxa than fungi. In fact, plants and protists were identified. Among *Protista*, protozoa were especially abundant. Toju et al. (2012) [18] had already expressed this risk of fungal ITS1 primers (ITS1-F_ KYO2 and ITS2_KYO2) matches with other eukaryotic sequences. ITS region is probably the most effective genetic marker and frequently used for fungi identification. However, its use can give limited results, namely because of its inter and intra-specific variability among fungi leading to weaker identification rates compared to bacteria [32,33].

In the PCoA plots, it could be noted that the inoculation of *P. aphanidermatum* produced a shift in microbiota β-diversity for most treatments in the rhizoplane. Although AP and AP-Pa are clustered differently, this shift was highly reduced for bacteria. This indicates a better resilience or resistance of the bacterial aquaponic microbiota to the entry of a perturbation, in this case, a plant pathogen. HP and CAP treatments induced a more disease conducive microbiota. The fact that bacterial diversity was quite similar after plant pathogen inoculation in suppressive environment is current in literature [34,35,36], while a disease conducive environment is more subject to bacterial modifications [35,37]. However, the resulting microbiota on diseased plants (i.e., after *P. aphanidermatum* inoculation) cannot help identifying antagonistic microorganisms by comparison between treatments. Indeed, for the correlation analysis, only healthy controls of AP, CAP, and HP were chosen to link α-diversity indexes or relative OTU abundances with suppressiveness indexes. The reason for this was that microbiota of diseased lettuce could result of post contamination of damaged/rotted tissues by opportunistic microorganisms masking the initial microbiota. Initial microbiota that was besides unable to control the pathogen entry in the case of CAP and HP treatments.

Initially, the presence of the CAP water treatment was added to counteract the potential bias in lettuce growth created by the lowest nutrients concentration in AP water compared to HP water. However, it was interesting to observe that CAP water (prepared from AP water) lost its potential disease suppressiveness by adding nutrient salts and lowered pH. Explanation could be linked to a microbiota modification as indicated in the α- and β-diversity and taxonomical composition of the rhizoplane. It was accorded that this type of parameters (e.g., pH and plant mineral nutrients) could impact microbiota diversity and also its suppressiveness [12]. Moreover, modification of water quality parameters could also have an indirect impact by playing on lettuce health or a direct impact on *Pythium* spp. development. In soil, Martin and Loper (1999) [20] reviewed the effect of pH on *Pythium* species. It was demonstrated in it that pH level impact disease development. Even if no general rules can be exposed, in some case, a pH under 7 can be linked to a better disease development on plant. This phenomena could be enlightened by a pH influence on zoospores production (in hydroponic system cropping lettuce in Funck-Jensen and Hockenhull (1983) [38]), on appressoria formation [39] cited by [4], on mycelial growth and on saprophytic activity of the pseudo-fungus reviewed by [20]. Furthermore, mineral or organic components (where composition and availability are also influenced by pH) of the environment can also influence *Pythium* spp. development [20,40,41]. In fact plant substrate or nutritive solution richer in nutrients can also sometimes enhance fungal diseases [42,43,44].

In the present study, higher α-diversity indexes were correlated with higher suppressiveness ability and more especially with species diversity where the relations were significant. In the literature about suppressive environments, this relation also appeared [37,45,46], but the reverse relation could be observed [47]. However, some of these references compared the microbiota of healthy plants and that resulting of ill plants where secondary or opportunistic microorganisms appear after the initial infection.

Among microorganism significantly correlated with suppressiveness in rhizoplane AP water in this study, *Lactobacillus* was the most known genus to be a plant disease antagonist, with *Lactobacillus plantarum* as key species [48,49]. *Sphingobium* genus was also present and found to be antagonist of *P. aphanidermatum* in Burgos-Garay et al. (2014) [50]. *Catenaria* genus is mainly known to be a nematodes antagonist [51,52]. However *Catenaria anguillulae* was also identified in Daft and Tsao (1984) [53] as parasite of *Phytophthora cinnamomi* and *parasitica;* two Oomycetes pathogens of citrus and avocado orchards. *Catenaria allomycis* was also described as fungus parasite on *Allomyces arbuscular* [54]. Rhizophydiales are, as *Catenaria* genus, member of Chytridomyces class. This class contains important parasite of invertebrates, protists and fungi. Notably, *Rhizophydium pythii*, who is a parasite of *Pythium* sp. [55] cited by [56]. An incertae sedis family belonging to *Hypocreales* was also correlated with disease suppression. Among this order, the well-known *Trichoderma* and *Gliocladium* genera could be cited for their antagonist activity against *Pythium* species [20]. The most abundant genus in this study correlated with suppressiveness was *Methyloversatilis*. Today, it is never related to plant disease suppression. This genus describes as methylotroph has the particularity to degrade variety of C1 units and multicarbon compounds, such as aromatic compounds, organic acids, alcohols, and methanol or methylamine [57,58]. After *Methyloversatilis*, the *Burkholderiaceae* family was the taxa most related to a suppressive action. Inside this family, the genus *Hydrogenophaga* was mentioned several times. *Burkholderiaceae* are linked to the bacterial composition of several suppressive soils [31,59]. This family contains several species able to act against fungal plant pathogens such as *Bulkhoderia* or *Mitsuaria* species [60], and notably against *P. aphanidermatum* [59]. The genus *Hydrogenophaga* is mainly known for its chemoorganotrophic or chemolithoautotrophic nutrition, using H_2_ as energy source and CO_2_ as a carbon source [61]. Moreover, it was cited and described as plant growth promotor essentially by acting in nitrogen cycle (denitrification and N_2_ fixation) [62].

HP water failed to support the inoculation of *P. aphanidermatum*. This difference with AP could be linked to a distinct microbiota composition and/or diversity in the rhizoplane, as well as difference of physicochemical water parameters (e.g., pH and mineral nutrients), these factors being closely related. This distinct and suppressive microbiota found in AP was probably driven by the presence of organic compounds in the nutritive solution. Stouvenakers et al. (2019) [1] envisaged this possibility by making links with the suppressiveness met in some farming systems containing higher rates of organic carbons (e.g., organic hydroponics). However, it was supported in the literature that hydroponic systems without organic amendments could also express a suppressive activity against plant pathogens reviewed by [7]. The explanation of why our HP water failed to suppress *P. aphanidermatum* disease development could be linked to an absence of system cycling before the experimentation. In fact the hypothesis behind the suppressive activity observed in hydroponics was the recirculated aspect of the nutrient water solution in the system [34,63,64] cited by [7]. This water recirculation created a stable and well-established microbiota after a certain period of cycling system.

In this in vivo study, growth of healthy lettuce was not compared between treatments but raw data of fresh leaf mass (data not shown) indicated a lower foliar yield of AP lettuce compared to HP and similar yield between CAP and HP. This could be logical because AP water contained lower concentration of mineral nutrients. However, this is in contradiction with other papers where yields of AP were similar to HP, and CAP yields better than AP [65,66,67,68,69,70]. In these papers, authors explained this contradiction (lower mineral nutrients concentrations but good yields) by the potential presence of microorganisms or compounds able to increase plant growth [71]. This hypothesis was strengthened by Sanchez et al. (2019) [72], who isolated several plant growth promoting bacteria in tilapia fish aquaculture. Explanations of the lower yield observed for AP lettuce in our experiment could be the bio-chemical variability of AP water or the stressing conditions applied to favor *P. aphanidermatum* infection that may impact development of plant growth promoting microorganisms.

## 5. Conclusions

Results of this study demonstrated that microorganisms of AP, RAS, and BM waters had a significant direct inhibitory effect on *P. aphanidermatum* growth in in vitro experiments. The suppressive effect of AP water was also shown on lettuce inoculated by *P. aphanidermatum* in in vivo conditions. Indeed, disease symptoms of AP lettuce were significantly reduced compared with CAP and HP lettuce. Root microbiota study suggested that AP water’s suppressive effect was namely induced by differences in terms of microorganism composition and diversity. Moreover, it was shown that CAP water lost the natural suppressive capacity of AP water after addition of nutrient salts and pH modification of AP water to create CAP water. Several microorganisms were significantly correlated with the suppressive effect of AP water. Nevertheless, few of these microorganisms (at the genus level) were known to have an antagonistic effect against *P. aphanidermatum*. In conclusion, these results indicated that AP water could be an interesting and novel source of antagonistic agents able to control *P. aphanidermatum* diseases in soilless culture.

## Figures and Tables

**Table 1:** Broth composition depending on the test and the modality for *P. aphanidermatum* inoculation.

	Modalities			
Test Name	Broth Composition with 25% of the Water Tested	Broth Composition with 25% of the Filtrated Water Tested		Positive Control Broth Composition
RAS *	15 mL of V8-75% + 5 mL of RAS water	15 mL of V8-75% + 5 mL of F-RAS water		V8
AP *	15 mL of V8-75% + 5 mL of AP water	15 mL of V8-75% + 5 mL of F-AP water		V8
BM *	15 mL of V8-75% + 5 mL of BM water			15 mL of V8-75% + 5 mL of KPBT
	

* The test was replicated thrice with different water samples taken within one week of interval and each replicated test was repeated thrice with the same water sample. RAS: recirculated aquaculture system, F-RAS: 0.2 µm filtrated recirculated aquaculture system, AP: aquaponic, F-AP: 0.2 µm filtrated aquaponic, BM: biofilter microbiota, V8: classical V8 CaCO3 broth, V8-75%: V8 CaCO3 broth containing only 75% of distilled water.

**Table 2:** Pearson correlation coefficients between species richness (observed_OTU number) or species diversity (Shannon index) of 16S rDNA or ITS analysis with suppressiveness indexes (F_f_MD, F_d_MD, FTD, and CRRR). Asterisks indicate statistically significant correlation by ANOVA (*p* ≤ 0.05).

	Correlation Coefficients with Suppressiveness Indexes			
	F_f_MD	F_d_MD	FTD	CRRR
**16S rDNA analysis**				
Species richness (Observed_OTU number)	−0.40	−0.24	−0.37	−0.55
Species diversity (Shannon index)	−0.83 *	−0.77 *	−0.83 *	−0.71 *
**ITS analysis**				
Species richness (Observed_OTU number)	−0.82 *	−0.57	−0.65	−0.78 *
Species diversity (Shannon index)	−0.86 *	−0.75 *	−0.84 *	−0.79 *

**Table 3:** Pearson correlation coefficients between taxa (OTU) relative abundances of 16S rDNA analysis and suppressiveness indexes (F_f_MD, F_d_MD, FTD, and CRRR). Only correlations with the 30 most abundant taxa in AP are reported (representing an abundance of 72.8%). Asterisks on OTU relative abundance indicate significant difference of abundance by Discrete False—Discovery Rate (DS-FDR) test (*p* ≤ 0.05) between treatments (AP, CAP, and HP water). Asterisks on suppressiveness indexes indicate statistically significant correlation by ANOVA (*p* ≤ 0.05).

Bacterial Taxa of Corresponding OTU and Their Mean Abundance in AP	Correlation Coefficients with Suppressiveness Indexes			
	F_f_MD	F_d_MD	FTD	CRRR
f_*Rhodocyclaceae*; g_*Methyloversatilis*: 8.02% *	−0.88 *	−0.62 *	−0.79 *	−0.95 *
f_*Burkholderiaceae*: 7.30% *	−0.89 *	−0.61 *	−0.75 *	−0.98 *
f_*Sphingomonadaceae*; g_*Sphingobium*: 5.61% *	−0.87 *	−0.61 *	−0.73 *	−0.95 *
f_*Microscillaceae*; g_*uncultured*: 5.01% *	−0.88 *	−0.61 *	−0.74 *	−0.97 *
f_*Streptococcaceae*; g_*Streptococcus*; s_*unculturedbact*: 3.97% *	−0.78 *	−0.43	−0.58 *	−0.89 *
f_*Lactobacillaceae*; g_*Lactobacillus*; s_*unculturedbact*: 3.87% *	−0.77 *	−0.45	−0.60 *	−0.86 *
f_*Pedosphaeraceae*; g_*unculturedbact*: 3.62% *	−0.88 *	−0.60 *	−0.73 *	−0.98 *
f_*Burkholderiaceae*: 3.51% *	−0.81 *	−0.55	−0.63 *	−0.84 *
f_*Sphingomonadaceae*; g_*Sphingobium*: 3.46% *	−0.79 *	−0.49	−0.65 *	−0.92 *
c_*Blastocatellia(Subgroup4)*; o_11–24; f_*unculturedbact*: 2.62% *	−0.90 *	−0.60 *	−0.75 *	−1.00 *
f_*Burkholderiaceae*: 2.62% *	−0.89 *	−0.62 *	−0.74 *	−0.97 *
f_*Burkholderiaceae*; g_*Hydrogenophaga*; s_*unculturedbact*: 2.46% *	−0.89 *	−0.61 *	−0.74 *	−0.97 *
f_*Burkholderiaceae*; g_*Hydrogenophaga*; s_*unculturedbact*: 2.17% *	−0.89 *	−0.61 *	−0.75 *	−0.98 *
c_*Gammaproteobacteria*; o_*CCD24*: 2.11% *	−0.88 *	−0.61 *	−0.74 *	−0.97 *
f_*Burkholderiaceae*: 1.77% *	−0.89 *	−0.61 *	−0.75 *	−0.98 *
f_*Lactobacillaceae*; g_*Lactobacillus*: 1.43% *	−0.87 *	−0.55	−0.66 *	−0.94 *
f_*Hyphomicrobiaceae*; g_*Hyphomicrobium*: 1.36% *	−0.82 *	−0.53	−0.67 *	−0.94 *
*f_*Nitrosomonadaceae: 1.19% *	−0.87 *	−0.59 *	−0.75 *	−0.97 *
f_*Nitrosomonadaceae*; g_*MND1*; s_*unculturedbact*: 1.17% *	−0.85 *	−0.58 *	−0.73 *	−0.94 *
f_*Saprospiraceae*; g_*uncultured*; s_*unculturedbact*: 1.17% *	−0.82 *	−0.56	−0.71 *	−0.93 *
f_*Chromobacteriaceae*; g_*Vogesella*; s.*unculturedbact*: 1.06% *	−0.81 *	−0.58 *	−0.65 *	−0.87 *
f_*Fimbriimonadaceae*: 0.99% *	−0.85 *	−0.54	−0.73 *	−0.96 *
f_*Propionibacteriaceae*; g_*Propionibacterium*: 0.89% *	−0.75 *	−0.48	−0.61 *	−0.81 *
f_*Gemmataceae*; g_*uncultured*; s_*unculturedbact*: 0.89% *	−0.81 *	−0.53	−0.71 *	−0.93 *
f_*Methylophilaceae*; g_*Methylophilus*; s_*unculturedbact*: 0.82%	−0.56	−0.52	−0.51	−0.46
f_*Reyranellaceae*; g_*Reyranella*: 0.79% *	−0.84 *	−0.57	−0.72 *	−0.95 *
f_*Lactobacillaceae*; g_*Lactobacillus*: 0.79% *	−0.82 *	−0.52	−0.63 *	−0.86 *
f_*Burkholderiaceae*: 0.71% *	−0.90 *	−0.62 *	−0.75 *	−0.98 *
f_*Sphingomonadaceae*: 0.71% *	−0.86 *	−0.57	−0.74 *	−0.96 *
f_*Nocardiaceae*; g_*Rhodococcus*; *Ambiguous_taxa*: 0.71% *	−0.28	−0.53	−0.48	0.08

**Table 4:** Pearson correlation coefficients between taxa (OTU) relative abundances of 16S rDNA analysis and suppressiveness indexes (F_f_MD, F_d_MD, FTD, and CRRR). Only correlations with the 30 most abundant taxa in AP are reported (representing an abundance of 83.0%). Unassigned OTUs and assignations limited to Fungi Kingdome were manually blasted and indicated for information in italic. Asterisks on OTU relative abundance indicate significant difference of abundance by DS-FDR test (*p* ≤ 0.05) between treatments (AP and HP). Asterisks on suppressiveness indexes indicate statistically significant correlation by ANOVA (*p* ≤ 0.05). CAP treatment is not part of the correlation analysis because it was removed by the rarefaction process during bioinformatics.

Bacterial Taxa of Corresponding OTU and Their Mean Abundance in AP	Correlation Coefficients with Suppressiveness Indexes			
	F_f_MD	F_d_MD	FTD	CRRR
k_*Fungi; uncultured*: 13.91%	−0.50	−0.11	−0.45	−0.64
k_*Fungi*; f_*Debaryomycetaceae*;*g_Meyerozyma*: 13.16%	0.54	0.78 *	0.80 *	0.27
k_*Fungi*: 9.30%	−0.50	−0.33	−0.42	−0.50
*k_Fungi*; f_*Catenariaceae*;g_*Catenaria*;s_unidentified: 4.70% *	−0.77 *	−0.36	−0.68	−0.90 *
k_*Fungi;* uncultured: 4.30% *	−0.67	−0.32	−0.63	−0.78 *
k_*Fungi;* uncultured: 4.09% *	−0.82 *	−0.44	−0.70	−0.90 *
k_*Fungi*; f_*Catenariaceae*;g_*Catenaria*;s_unidentified: 3.79% *	−0.79 *	−0.33	−0.71 *	−0.94 *
k_*Protista;* c_*Kinetoplastida*: 3.70% *	−0.70	−0.26	−0.63	−0.85 *
k_*Protista;* c_*Kinetoplastida*: 2.98% *	−0.68	−0.30	−0.60	−0.82 *
k_*Fungi* o_*Rhizophydiales*: 2.76% *	−0.76 *	−0.41	−0.69	−0.85 *
k_*Fungi;* uncultured: 2.64%	−0.16	−0.11	−0.23	−0.22
k_*Protista;* g_*Trypanosoma*: 1.79%	−0.76 *	−0.51	−0.75 *	−0.73 *
k_*Protista;* g_*Trypanosoma*: 1.52%	−0.73 *	−0.40	−0.68	−0.75 *
k_*Fungi;* uncultured: 1.47% *	−0.65	−0.20	−0.63	−0.83 *
k_Fungi; o_Dothideales: 1.38%	−0.43	−0.29	−0.34	−0.36
k_*Fungi;* uncultured: 1.08% *	−0.81 *	−0.37	−0.73 *	−0.95 *
k_*Fungi;* uncultured: 1.05% *	−0.81 *	−0.40	−0.72 *	−0.92 *
k_*Fungi;* g_*Cladosporium*: 1.01%	−0.44	−0.31	−0.34	−0.37
k_*Fungi;* uncultured: 0.95%	−0.05	−0.45	−0.15	0.13
k_*Protista;* g_*Trypanosoma*: 0.93% *	−0.71	−0.40	−0.66	−0.77 *
k_*Viridiplantae; Embryophyta*: 0.89% *	−0.80 *	−0.41	−0.67	−0.86 *
k_*Protista;* g_*Trypanosoma*: 0.82% *	−0.76 *	−0.51	−0.68	−0.73 *
k_Fungi; o_*Ustilaginales*: 0.77%	−0.35	−0.22	−0.27	−0.32
k_Fungi; f_*Ustilaginaceae*: 0.75%	−0.49	−0.32	−0.31	−0.42
k_*Fungi*; o_*Pleosporales*: 0.64%	−0.26	−0.15	−0.29	−0.26
k_*Fungi;* g_*Cladosporium*: 0.62%	−0.14	−0.11	−0.17	−0.07
f_*Fungi;* p_*Ascomycota; Pezizomycotina*: 0.52%	−0.40	−0.16	−0.45	−0.53
k_*Fungus*: 0.51%	−0.68	−0.34	−0.72 *	−0.74 *
k_*Fungi*; f_Aspergillaceae: 0.48%	−0.57	−0.05	−0.26	−0.72 *
k_Fungi; f_Hypocreales_fam_Incertae_sedis: 0.46% *	−0.85 *	−0.49	−0.74 *	−0.92 *

## Supplementary Materials

The following are available online at https://www.mdpi.com/2076-2607/8/11/1683/s1.

## Abbreviations

ANOVA	analysis of variance
AP	aquaponic
AP-Pa	aquaponic plus *P. aphanidermatum*
BM	biofilter media
BOD5	biological oxygen demand in 5 days
CAP	complemented aquaponic
CAP-Pa	complemented aquaponic plus *P. aphanidermatum*
CFU	colony forming unit
CRRR	corrected root rot rating
DS-FDR	discrete false - discovery rate
Ec	electroconductivity
F-AP	filtrated aquaponic water
F_d_M	foliar dry mass
F_d_MD	foliar dry mass decrease
F_f_M	foliar fresh mass
F_f_MD	foliar fresh mass decrease
F-RAS	filtrated recirculated aquaculture water
FTD	relative foliar turgidity decrease
FWC	foliar water content
HC	healthy control
HP	hydroponic
HP-Pa	hydroponic plus *P. aphanidermatum*
IL	inoculated
ITS	internal transcript spacer
KPBT	kalium phosphate buffer and tween
LB	Luria-Bertani
OTU	operational taxonomic unit
PAR	photosynthetically active radiation
PC1/2/3	principal coordinates 1/2/3
PCoA	principal coordinates analysis
PDA	potatoes dextrose agar
PERMANOVA	permutational multivariate analysis of variance
q2	Qiime 2
RAS	recirculated aquaculture system
rDNA	ribosomal DNA
RRR	root rot rating
