# Supplementary material for: Microbial Origin of Aquaponic Water Suppressiveness against Pythium aphanidermatum Lettuce Root Rot Disease
Source: Microorganisms. 2020 Oct 29;8(11):1683. doi: 10.3390/microorganisms8111683 (PMC7694120; doi:10.3390/microorganisms8111683)
Supplement: Supplementary file 1 [file microorganisms-08-01683-s001.pdf]

microorganisms

Supplementary Materials

S1. Water origin

For the *in vitro* tests, 3 different types of water were tested independently, i.e. recirculated aquaculture system (RAS) water, aquaponic (AP) water and washing water containing microorganisms of the AP biofilter media (BM for Biofilter Microbiota). For the *in vivo* test, AP water, complemented aquaponic (CAP) water and hydroponic (HP) water were tested. The RAS system is described in supplementary materials of Eck et al., 2019 [1]. Briefly it consists in a Nile Tilapia (Oreochromis niloticus) fish husbandry kept in 3 tanks of 380 litres at a density of 60kg/m3, a drum filter to remove sludge, a biofilter containing Biocerapond media to transform ammonia to nitrate, and a sump where water is pumped to go back in the fish tanks. The AP system named PAFF Box for Plant And Fish Farming Box is fully explained in Delaide et al., 2017 [2]. This system is composed of 2 tanks of 380 litres at a density of 60kg of Nile Tilapia per m3, a lamellar settler to remove solids (upgrade from [2]), a sump where water is pumped through a microbeads biofilter (SHARK BEAD 45/25) and then goes upstairs in a greenhouse to supply a raft hydroponic system (deep water culture) composed of a majority of lettuces with other additional plants (basil, swiss chard, strawberries, parsley, coriander, cucumber, hot pepper, tomatoes and watercress). Fish in RAS and in AP system were fed with adapted level of tilapia feed (TI-4.5 Tilapia 4.9mm, Skretting, Fontaine Les Vervins, France). RAS and AP water samples were taken respectively in the RAS and in PAFF Box sump. The washing water with the BM was recovered as followed: centrifuge tubes of 50ml filled with 15g of microbead from the PAFF Box SHARK BEAD biofilter were sonicated in ultrasound bath during 10min with 30ml of 0.05M Kalium Phosphate Buffer plus 0.05% Tween 80 (KPBT) at pH 6,5. The washing solution containing microorganisms scraped off from the microbeads was filtrated in a cheese cloth to remove microbeads and biggest particles and then used for the experiment. CAP water is the water taken in the PAFF Box sump where mineral salts were added to reach hydroponic nutrients concentration. Finally, HP water is a hydroponic nutrient solution made by addition of mineral salts in demineralized water. For the *in vivo* test, composition of AP, CAP and HP water were summarized in Table S1.

Table S1: Bio-chemical parameters of aquaponic (AP), hydroponic with half nutrient salts (½ HP), hydroponic (HP) and complemented aquaponic (CAP) waters for the 2 tests replication (trial 1 and 2). The parameters measured were BOD5, microorganisms concentration, pH, Ec, and mineral nutrients concentration.

Water kind	BOD5 in mg/l	Microorganisms concentration in CFU/ml		pH	Ec in µS/cm	Macronutrients in mg/l							Micronutrients in mg/l					
		PDA dishes	LB dishes			NO_3^--N	NH_3^+-N	PO_4^{3-}-P	K^+	Ca^{2+}	Mg^{2+}	SO^{4-}-S	Fe^{2+}	Zn	B	Mn	Cu	Mo
AP Trial 1	6.0	$0.9x10^3$	$9.0x10^3$	7.83	1380[y]	65.5	0.7	32.4	30.0	170.0	35.0	48.5	0.00	NM	NM	NM	NM	NM
AP Trial 2	4.6	$2.5x10^4$	$8.4x10^4$	7.38	1200[y]	43.3	0.8	25.0	90.0	100.0	30.0	66.7	0.12	NM	NM	NM	NM	NM
½ HP Trial 1	NM	$1.1x10^4$	$1.5x10^4$	5.72[z]	1161[x]	82.6	12.7	25.0	98.3	100.0	20.0	40.8	0.00	0.00	0.00	0.00	0.00	0.00
½ HP Trial 2	NM	$1.9x10^5$	$3.4x10^5$	5.57[z]	1083[x]	82.6	12.7	25.0	98.3	100.0	20.0	40.8	0.00	0.00	0.00	0.00	0.00	0.00
HP Trial 1	NM	NM	NM	5.67[z]	2210[x]	164.9	25.1	50.0	210.0	200.1	40.0	87.5	5.00	0.10	0.50	0.50	0.10	0.03
HP Trial 2	NM	NM	NM	5.64[z]	1971[x]	164.9	25.1	50.0	210.0	200.1	40.0	87.5	5.00	0.10	0.50	0.50	0.10	0.03
CAP[w] Trial 1	NM	NM	NM	5.77[z]	2515[y]	161.5	29.8	68.2	211.5	202.9	44.74	48.9	5.00	0.10	0.50	0.50	0.10	0.05
CAP[w] Trial 2	NM	NM	NM	5.72[z]	2245[y]	165.0	25.0	50.0	210.0	200.0	40.0	90.4	5.00	0.10	0.50	0.50	0.10	0.05

[w]The first week, CAP waters had the same composition than AP water with a pH adjusted to 5.5 - 5.8.

[x]Ec of ½ HP and HP waters differ between the trial 1 and 3 due to a different demineralized water quality (not measured).

[y]The organic matter and the non-linear NaCl concentration of AP water can disturb Ec measures.

[z]pH was measured after the first pH adjustment to the range 5.5 - 5.8

Sulfate was used as degree of freedom

NM = not measured

S2. Root microbiota taxonomical composition

S2.1. 16S rDNA endosphere

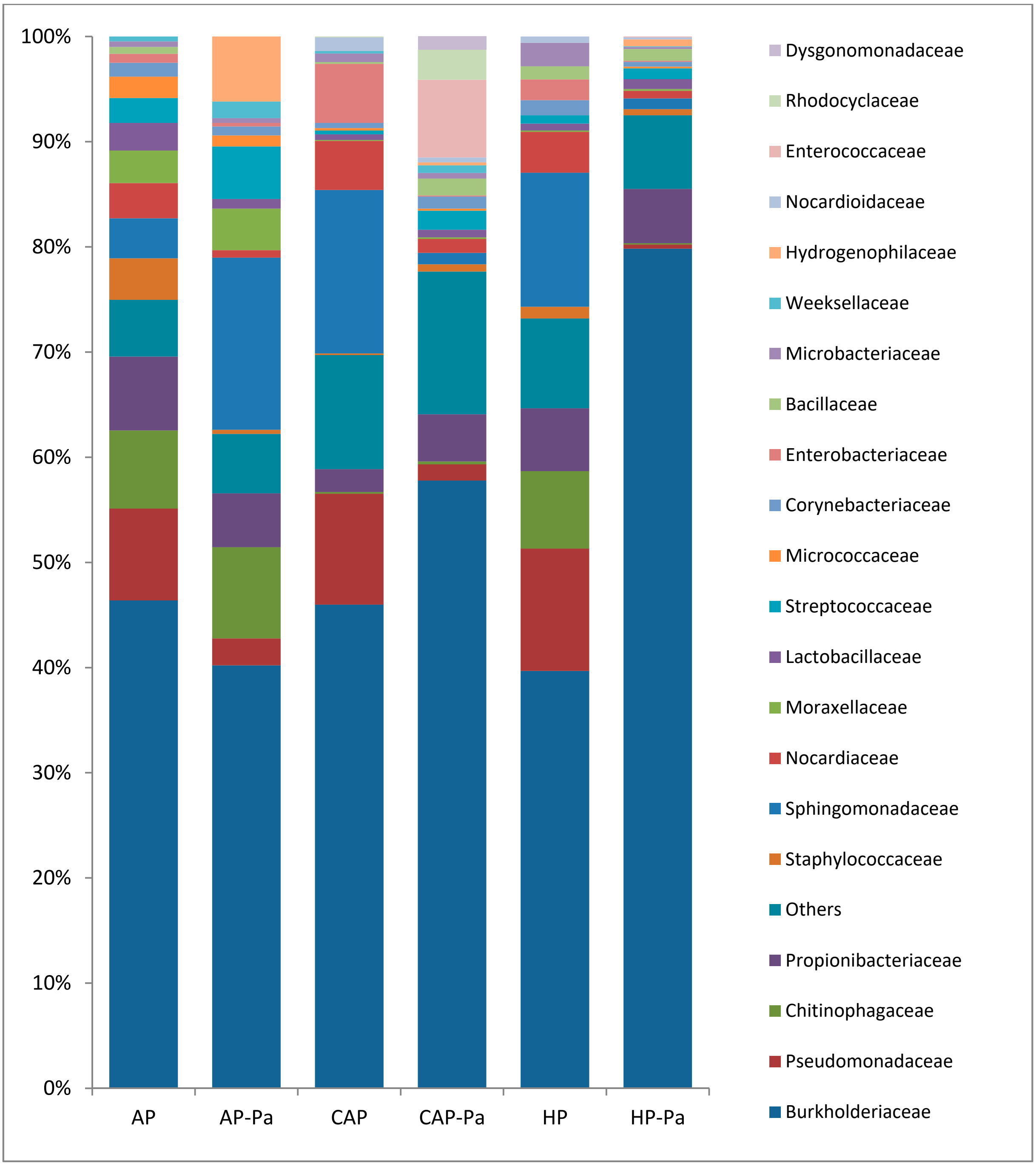

Figure S1. Means of bacterial relative composition, at family level, of lettuces roots endosphere depending on the treatment. Treatments are aquaponic (AP), hydroponic (HP) and complemented aquaponic (CAP) waters and respectively AP-Pa, HP-Pa and CAP-Pa water after lettuces inoculation with *P. aphanidermatum*. Only the OTUs with proportion higher than 1% were represented and the rest were clustered in “Others”.

S2.2. 16S rDNA rhizoplane

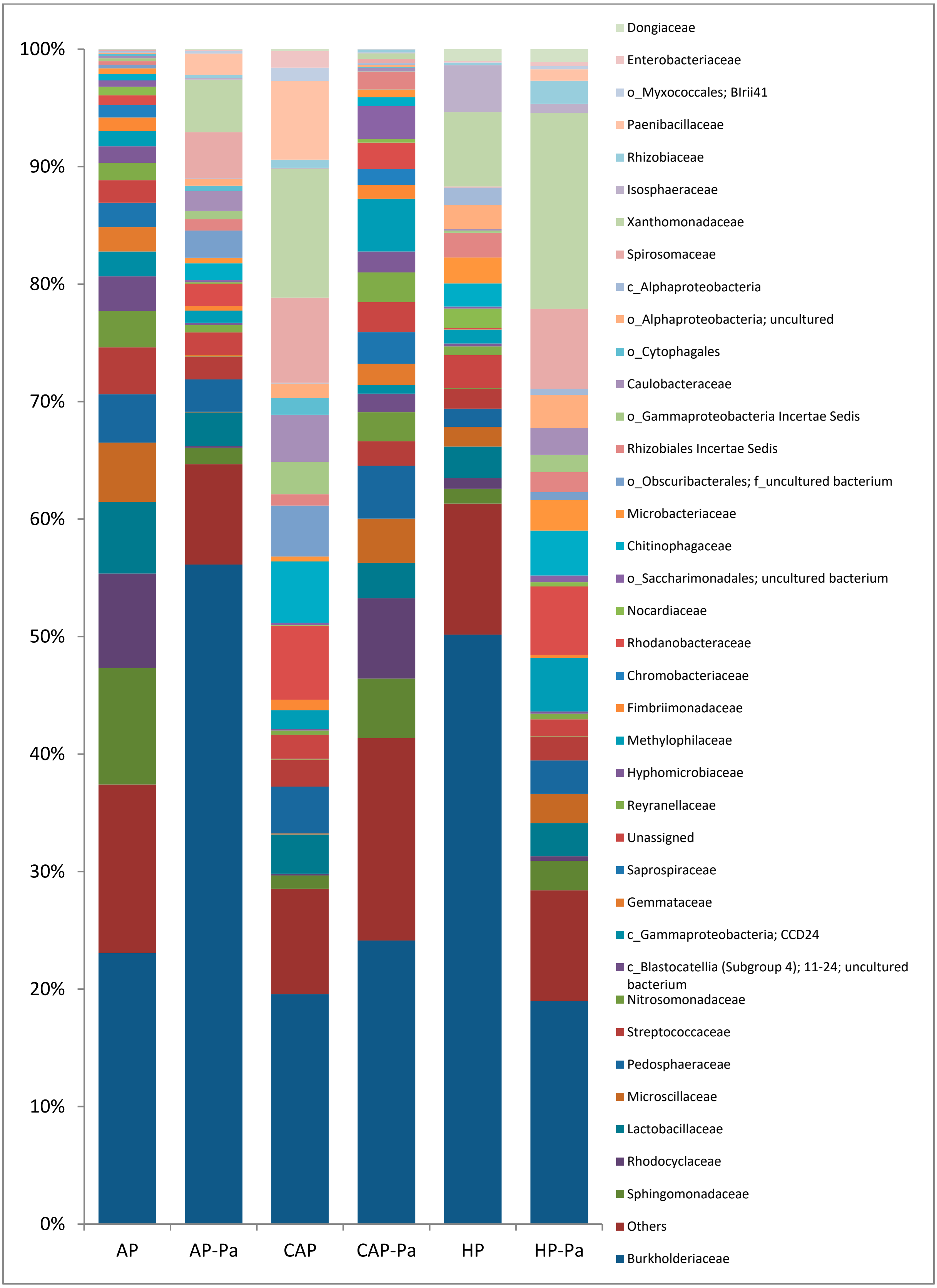

Figure S2. Means of bacterial relative composition, at family level, of lettuces roots rhizoplane depending on the treatment). Treatments are aquaponic (AP), hydroponic (HP) and complemented aquaponic (CAP) waters and respectively AP-Pa, HP-Pa and CAP-Pa water after lettuces inoculation

with *P. aphanidermatum*. Only the OTUs with proportion higher than 1% were represented and the rest were clustered in “Others”.

S2.3. *16S rDNA rhizosphere*

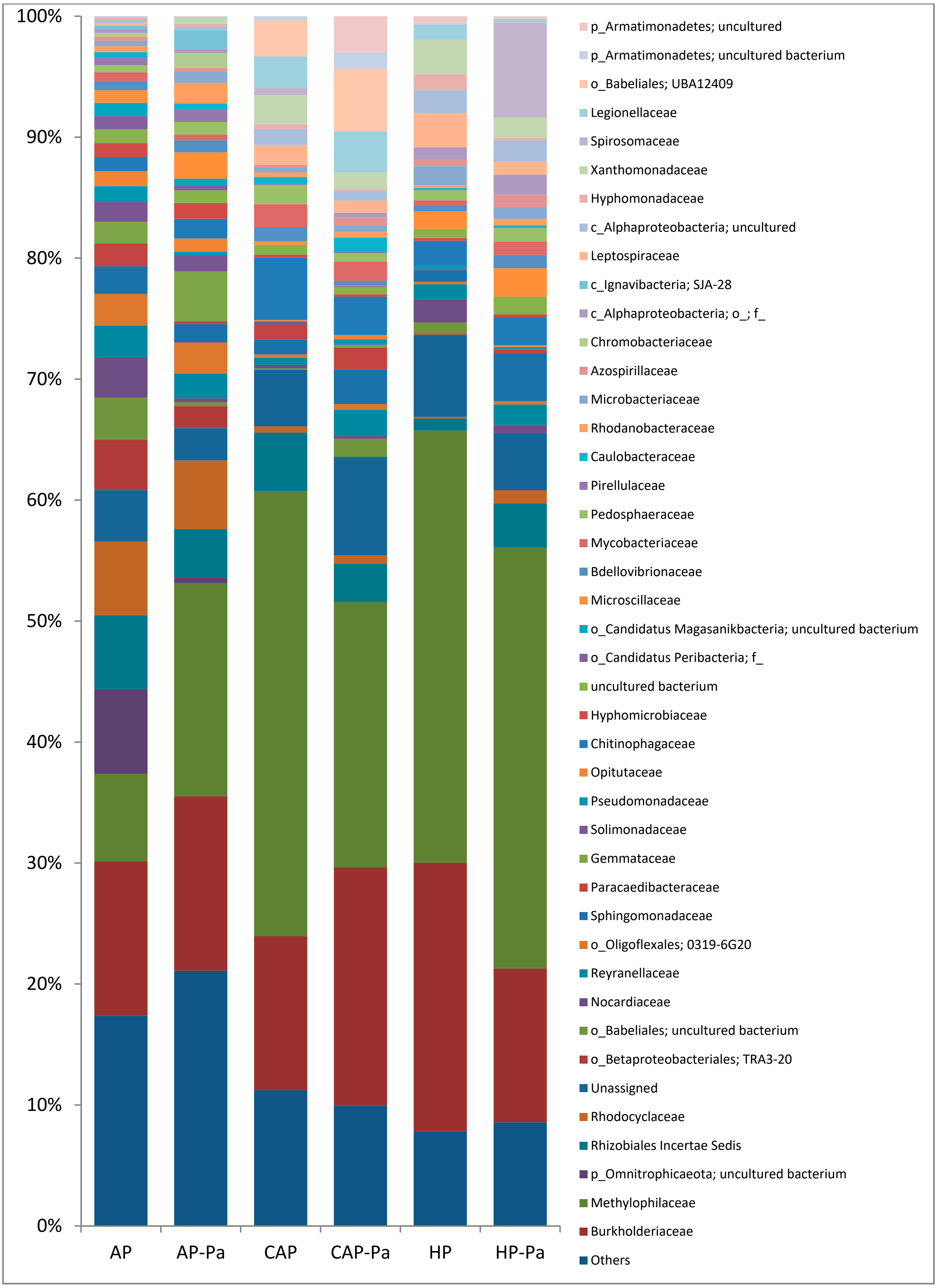

Figure S3. Bacterial relative composition, at family level, of lettuces roots rhizosphere depending on the treatment. Treatments are aquaponic (AP), hydroponic (HP) and complemented aquaponic (CAP) waters and respectively AP-Pa, HP-Pa and CAP-Pa water after lettuces inoculation with *P. aphanidermatum*. Only the OTUs with proportion higher than 1% were represented and the rest were clustered in "Others". Compositions were based on a unique microbiota sample

S2.4. *ITS endosphere*

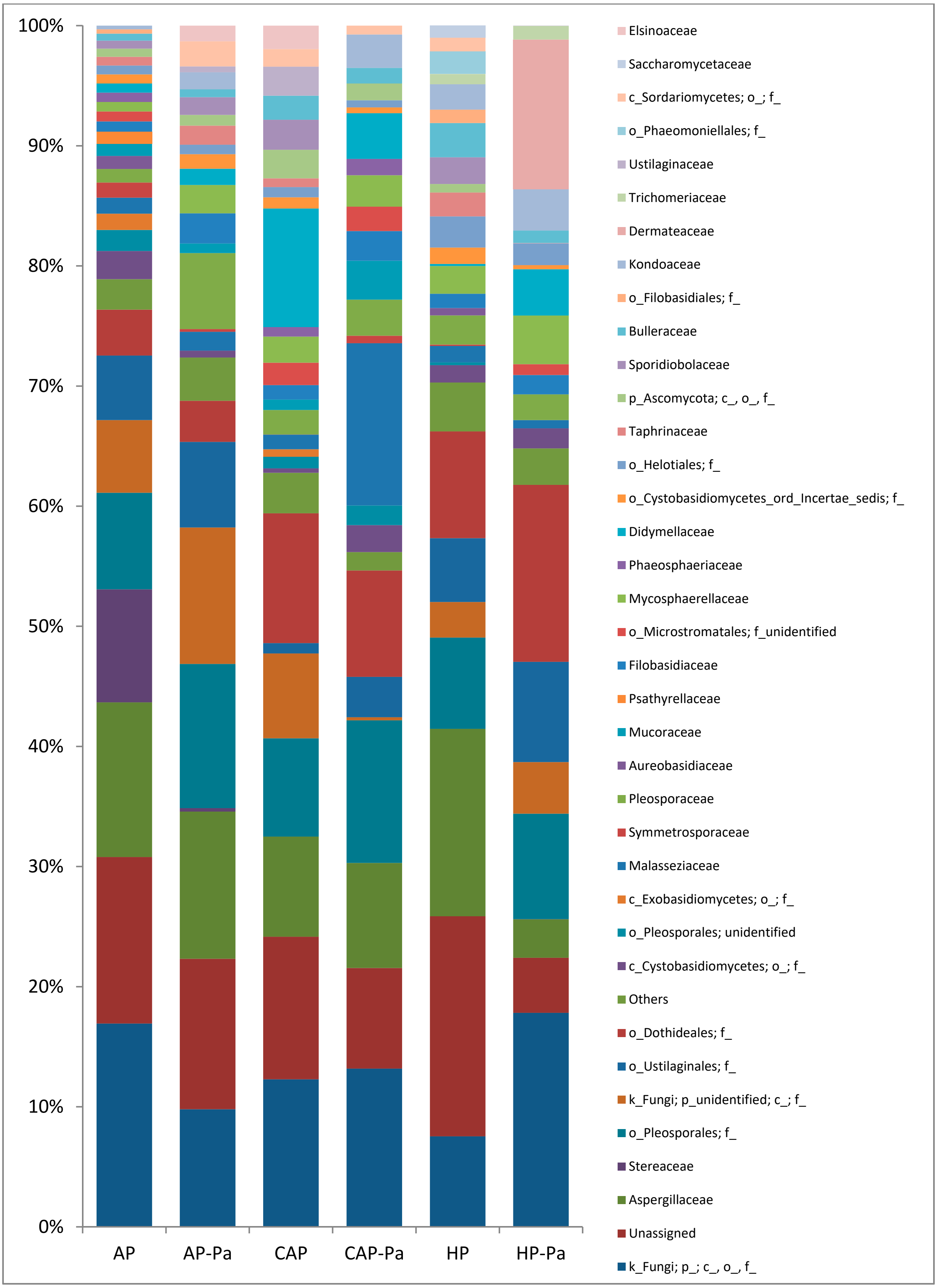

Figure S4. Means of fungal relative composition, at family level, of lettuces roots endosphere depending on the treatment. Treatments are aquaponic (AP), hydroponic (HP) and complemented aquaponic (CAP) waters and respectively AP-Pa, HP-Pa and CAP-Pa water after lettuces inoculation

with *P. aphanidermatum*. Only the OTUs with proportion higher than 1% were represented and the rest were clustered in “Others”.

S2.5. ITS rhizoplane

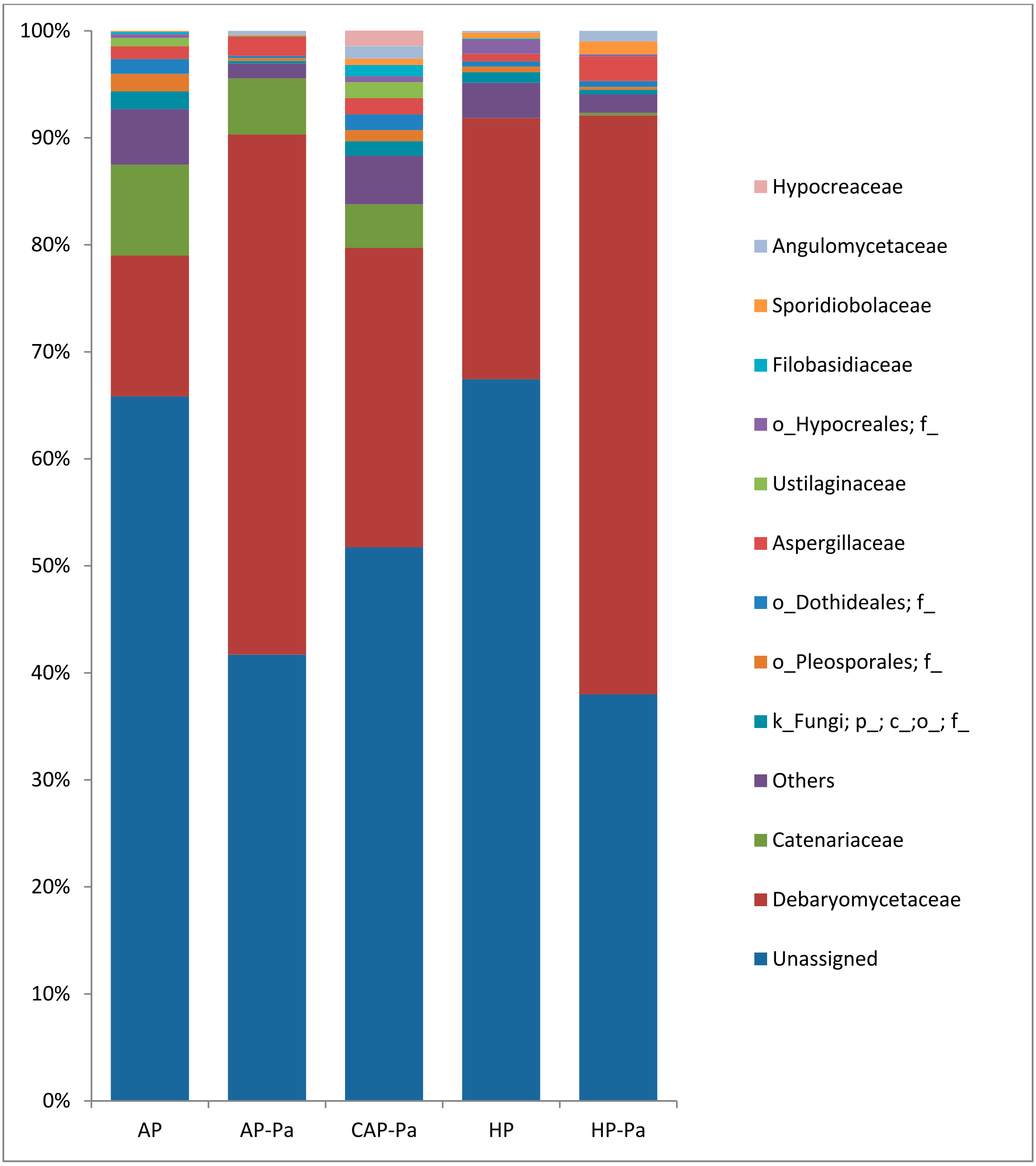

Figure S5. Means of fungal relative composition, at family level, of lettuces roots rhizoplane depending on the treatment). Treatments are aquaponic (AP), hydroponic (HP) and complemented aquaponic (CAP) waters and respectively AP-Pa, HP-Pa and CAP-Pa water after lettuces inoculation with *P. aphanidermatum*. Only the OTUs with proportion higher than 1% were represented and the rest were clustered in “Others”. CAP water was removed from the analysis during de bioinformatics process.

S2.6. ITS rhizosphere

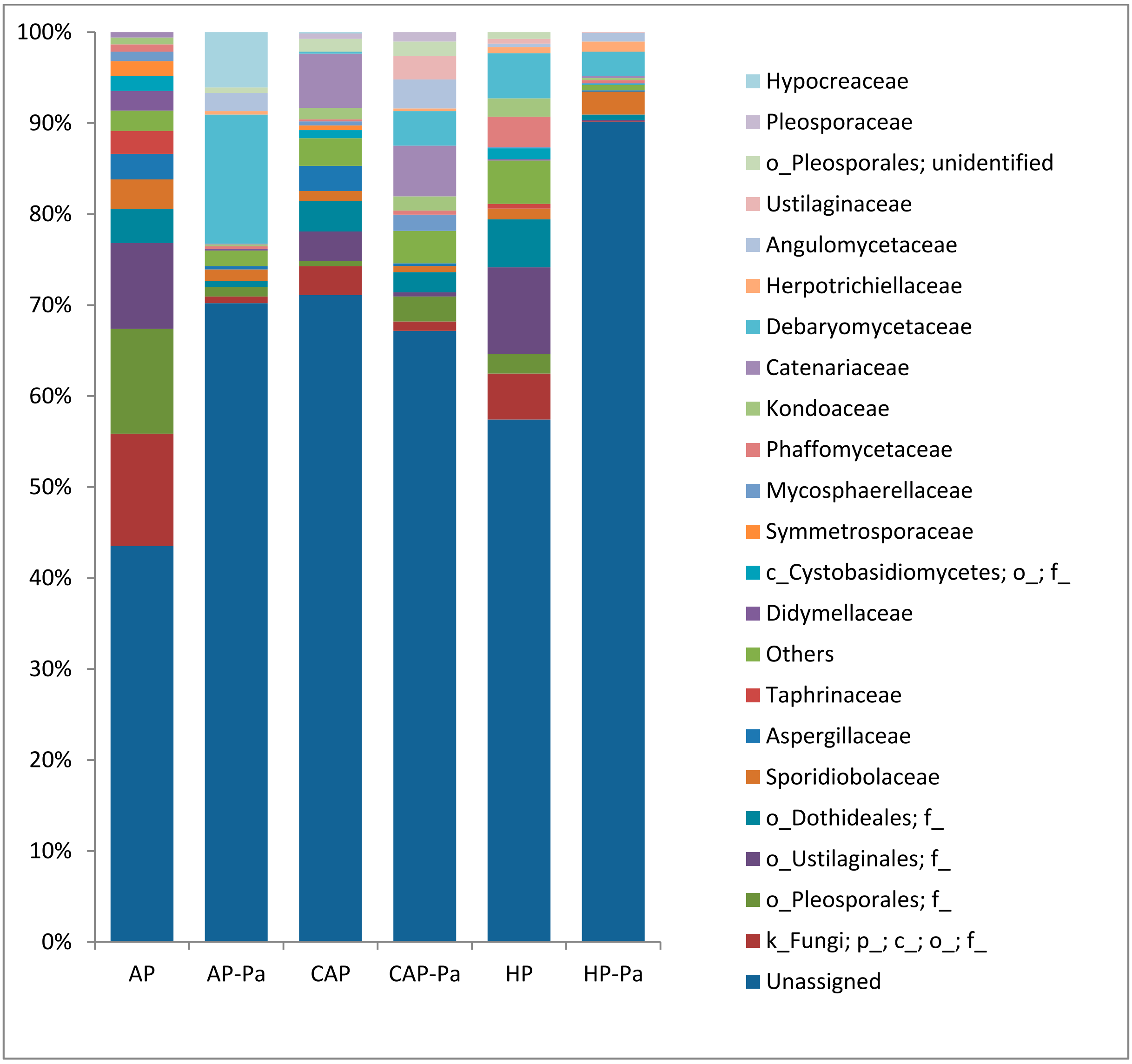

Figure S6. Fungal relative composition, at family level, of lettuces roots rhizosphere depending on the treatment. Treatments are aquaponic (AP), hydroponic (HP) and complemented aquaponic (CAP) waters and respectively AP-Pa, HP-Pa and CAP-Pa water after lettuces inoculation with *P. aphanidermatum*. Only the OTUs with proportion higher than 1% were represented and the rest were clustered in "Others". Compositions were based on a unique microbiota sample.

S3. Microbiota α-diversity

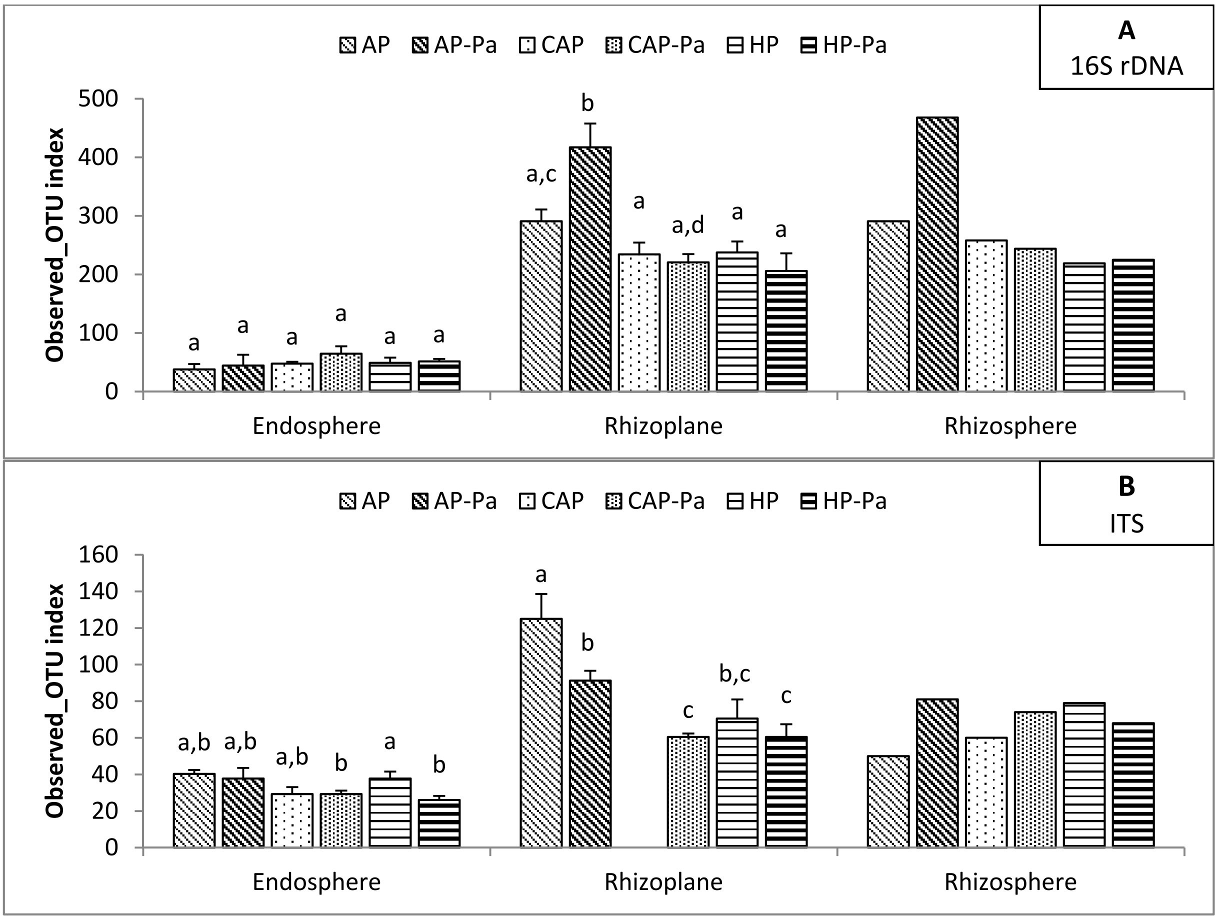

Figure S7. Species richness (observed_OTU number) of lettuces endosphere, rhizoplane and rhizosphere of (A) 16S rDNA and (B) ITS analyses depending on the treatment. Treatments are aquaponic (AP), hydroponic (HP) and complemented aquaponic (CAP) waters and respectively AP-Pa, HP-Pa and CAP-Pa water after lettuces inoculation with *P. aphanidermatum.* Bars indicate the standard error of the mean Treatments that do not share a same letter are significantly different by Kruskal-Wallis pairwise test ($p \leq 0.05$). CAP treatment in the ITS rhizoplane was removed by the rarefaction process during bioinformatic analysis. Rhizosphere microbiota was constituted of a unique sample by treatment and was not subject to statistical analysis.

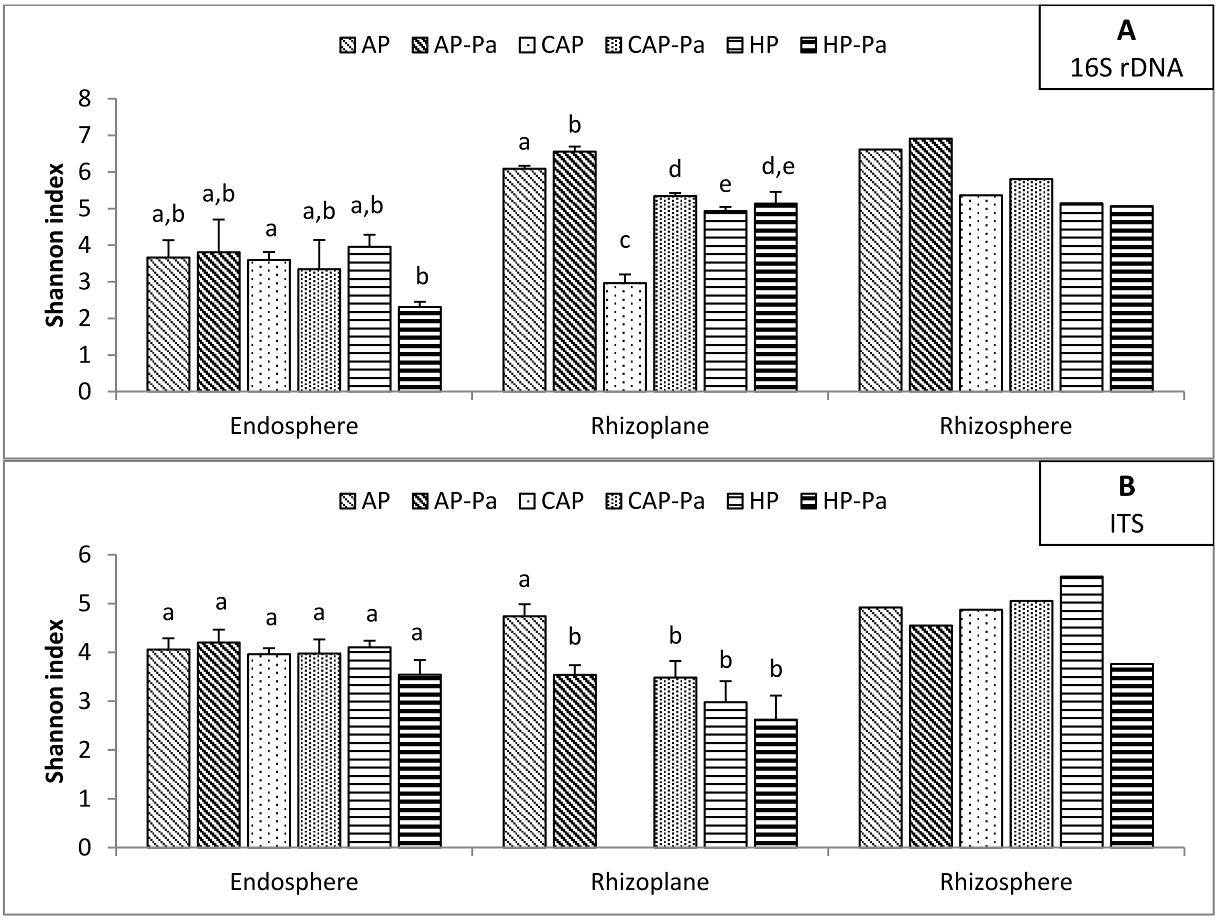

Figure S8. Species diversity (Shannon index) of lettuces endosphere, rhizoplane and rhizosphere of (A) 16S rDNA and (B) ITS analyses depending on the treatment. Treatments are aquaponic (AP), hydroponic (HP) and complemented aquaponic (CAP) waters and respectively AP-Pa, HP-Pa and CAP-Pa water after lettuces inoculation with *P. aphanidermatum*. Bars indicate the standard error of the mean. Treatments that do not share a same letter are significantly different by Kruskal-Wallis pairwise test ($p \leq 0.05$). CAP treatment in the ITS rhizoplane was removed by the rarefaction process during bioinformatic analysis. Rhizosphere microbiota was constituted of a unique sample by treatment and was not subject to statistical analysis.

References

1. Eck, M.; Sare, A.R.; Massart, S.; Schmautz, Z.; Junge, R.; Smits, T.H.M.; Jijakli, M.H. Exploring Bacterial Communities in Aquaponic Systems. *Water* **2019**, *11*, 1–16, doi:10.3390/w11020260.
2. Delaide, B.; Delhaye, G.; Dermience, M.; Gott, J.; Soyeurt, H.; Jijakli, M.H. Plant and fish production performance, nutrient mass balances, energy and water use of the PAFF Box, a small-scale aquaponic system. *Aquac. Eng.* **2017**, *78*, 130–139, doi:10.1016/j.aquaeng.2017.06.002.
